# Stabilized Chiral Organic Material Containing BINAP Oxide Units as a Heterogeneous Asymmetric Organocatalyst for Allylation of Aldehydes

**DOI:** 10.1021/acsami.3c04430

**Published:** 2023-06-12

**Authors:** Miguel Sánchez-Fuente, Alberto López-Magano, Alicia Moya, Rubén Mas-Ballesté

**Affiliations:** † Department of Inorganic Chemistry (Module 7), Facultad de Ciencias, Universidad Autónoma de Madrid, 28049 Madrid, Spain; ‡ Institute for Advanced Research in Chemical Sciences (IAdChem), Universidad Autónoma de Madrid, 28049 Madrid, Spain

**Keywords:** asymmetric catalysis, organocatalysis, chiral organic materials, phosphine oxides, aldol reaction

## Abstract

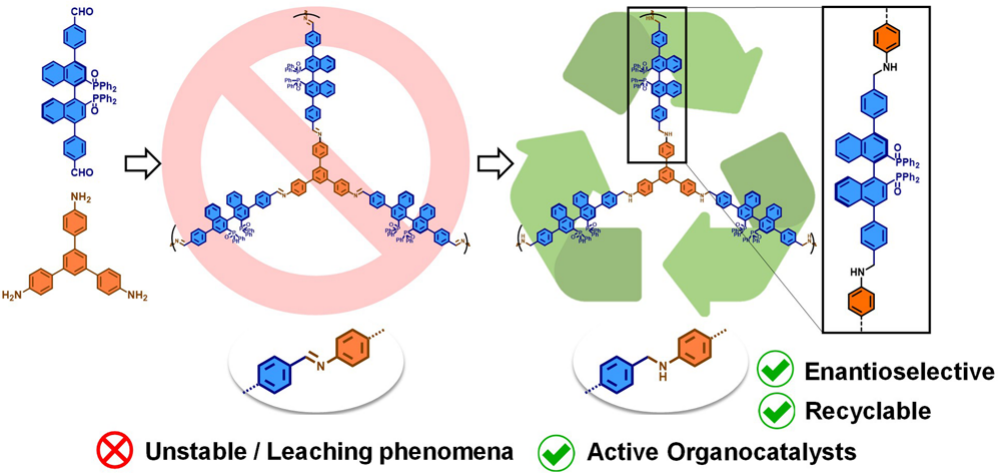

Condensation of BINAPO-(PhCHO)_2_ and 1,3,5-tris(4-aminophenyl)benzene (TAPB) results in a new imine-based chiral organic material (COM) that can be further post-functionalized through reductive transformation of imine linkers to amines. While the imine-based material does not show the necessary stability to be used as a heterogeneous catalyst, the reduced amine-linked framework can be efficiently employed in asymmetric allylation of different aromatic aldehydes. Yields and enantiomeric excesses found are comparable to those observed for the molecular BINAP oxide catalyst, but importantly, the amine-based material also permits its recyclability.

## 1 Introduction

Asymmetric organocatalysis has experimented an outstanding blossoming during the last few years because it enables effective synthetic routes for important enantiomerically pure products, avoiding the use of expensive or toxic metals.^1^ However, the limited stability of common catalysts and their difficult recyclability appear as some of their main limitations. Therefore, the quest for recyclable asymmetric organocatalytic systems is an important target in the field of heterogeneous catalysis.^2,3^ Toward this goal, an emerging strategy consists of incorporating asymmetric organocatalytic building blocks on extended organic materials such as porous aromatic frameworks (PAFs),^4^ covalent organic frameworks (COFs),^5^ and other porous organic polymers (POPs).^6^ Design of reticular materials offers many possibilities for the assembly of complex organic fragments into organized frameworks that display exceptional features, such as well-defined channels and tunability of chemical composition. Therefore, COFs and their amorphous counterparts have increasingly shown diverse applications as heterogeneous catalysts.^7−9^ However, enantioselective organocatalytic reactions catalyzed by chiral reticular organic materials are a field in its infancy. To date, reported synthetic strategies inducing chirality in such materials are the following: (a) direct polymerization of chiral organic monomers;^10,11^ (b) post-synthetic decoration of the preformed frameworks by chiral fragments;^12^ (c) chiral induction by asymmetric additives involved in the polymerization;^13^ and (d) assembly of organic frameworks through asymmetric catalysis.^14^ Although all these methodologies offer their advantages and drawbacks, when chiral building blocks are available, their direct polymerization allows their homogeneous distribution over the material’s framework.

**Figure 1:**
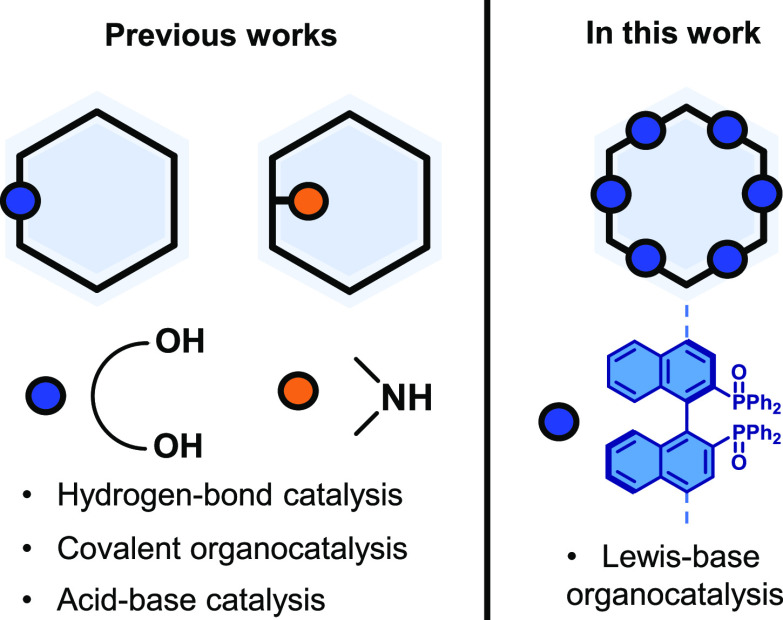
Related reported approaches and general idea of this work.

Despite the versatility of strategies that have been developed, only a limited range of chiral organocatalytic moieties has been incorporated into organic frameworks. In particular, BINOL,^15^ TADDOL,^10^ asymmetric pyrrolidines,^12^ propargylamines,^14^ and other chiral secondary amines have been to build chiral organocatalytic frameworks. Thus, there is a plethora of unexplored possibilities on the design of such heterogeneous catalytic systems. In the quest for incorporation of well-defined chiral single sites into organic frameworks, we designed a new material based on the assembly of a building block containing the axially chiral phosphine oxide BINAPO (Figure 1). Phosphine oxides show a high nucleophilicity resulting from the polarization in the P–O bond, which enables their role as Lewis bases in organocatalytic processes.^16^ Interestingly, one possible activation pathway mediated by phosphine oxides consists of the generation of hypervalent silicates from trichlorosilyl compounds, which can result in the formation of new C–C bonds. This feature has been already explored on homogeneous systems using BINAPO as a Lewis base catalyst for asymmetric allylation of aromatic aldehydes using trichlorosilyl derivatives.^17^ Owing to the interest in these transformations, we present a new BINAPO-containing chiral organic material (COM). The activities and selectivities observed for this new heterogeneous system rival those of its homogeneous analogs and allow a high degree of recyclability.

Most of reported chiral COFs are connected by imine linkages because of the availability of a wide range of functional building blocks and the efficiency of imine condensation for the assembly of extended frameworks.^18^ However, the reversible nature of imines grants only moderate chemical stability to the materials obtained.^19,20^ This factor hampers utility of imine-based COFs for their use as catalysts in the presence of reagents that could result in disassembly of the covalent framework such as the trichlorosilyl precursors used in this work. Fortunately, to still exploit the advantages of imine condensation, but at the same time avoid the problem of its limited sturdiness, some strategies for stabilization of imine linkages by post-functionalization processes have been reported.^21^ In this work, we present the reduction of imines in a BINAPO-containing COF-type structure to form the more stable amine linkages,^22^ which further enabled asymmetric organocatalysis in a recyclable manner. Overall, the asymmetric organocatalytic activities found in this new heterogeneous system open new perspectives on the design of organocatalytic materials with axial chirality.

## 2 Experimental Section

**Scheme 1:**
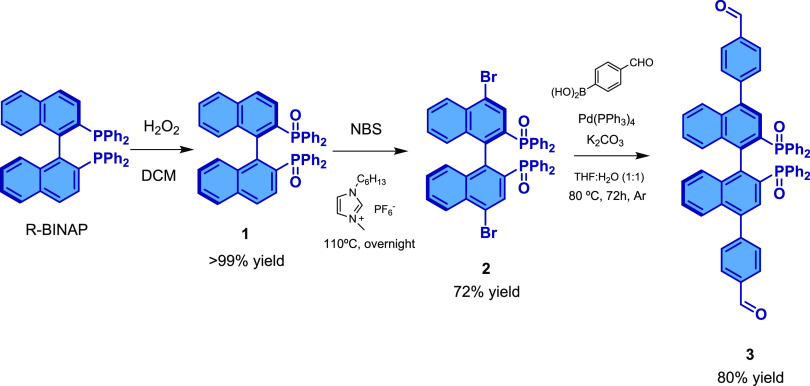
Synthetic Strategy for the Obtention of Building Block **3**

The synthetic strategy for the BINAPO-(PhCHO)_2_ building block (**3**) is presented in Scheme 1.

### 2.1 Synthesis of (*R*)-BINAPO (**1**)

In a 250 mL round flask equipped with a magnetic stirrer, commercially available (*R*)-BINAP (5 g, 8.03 mmol) was dissolved in dichloromethane (200 mL). Then, hydrogen peroxide (commercial 30% v/v solution in water, 25 mL) was added dropwise. The reaction progression was followed by TLC until the whole reagent was consumed (typically 4 h after the complete addition of the hydrogen peroxide). Once the reaction was finished, the crude mixture was washed with distilled water and extracted with dichloromethane and dried over Na_2_SO_4_ and the solvent was removed under reduced pressure. The (*R*)*-*BINAP oxide product (**1** in Scheme 1) was obtained as a white solid in >99% yield. All the experimental data obtained for the characterization of **1** agree well with data previously reported.^23^

^1^H-NMR (300 MHz, CDCl_3_): δ 7.86–7.80 (m, 4H), 7.73–7.66 (m, 4H), 7.46–7.36 (m, 12H), 7.28–7.24 (m, 8H), 6.80 (d, *J* = 2.8 Hz, 4H); ^31^P-NMR (400 MHz, CDCl_3_): δ 29.16; MALDI-TOF-MS: 655.3 (M + H)^+^, 677.2 (M + Na)^+^; Elemental analysis: theorical (C_44_H_32_O_2_P_2_·0.5H_2_O) % C 79.63, % H 5.01; exp.: % C 79.7, % H 5.3.

### 2.2 Synthesis of (*R*)-(4,4′-Dibromo-[1,1′-binaphthalene]-2,2′-diyl)bis(diphenylphosphine oxide) (**2**)

In a sealed 100 mL round Schlenk flask equipped with a magnetic stirrer, the ionic liquid 1-butyl-3-methylimidazolium hexafluorophosphate (25 g) was activated under vacuum at 80 °C under stirring for 4 h. Then, (*R*)-BINAP oxide **1** (2 g, 3.05 mmol) was added, followed by portion-wise addition of *N*-bromosuccinimide (1.9 g, 10.7 mmol). The crude mixture was heated to 110 °C under air overnight. Then, the mixture was cooled to room temperature and extracted with dichloromethane. The organic layers were combined and dried over Na_2_SO_4_, and the solvent was removed under reduced pressure. Then, 30 mL of cold ethanol was added to the mixture and it was let in the fridge to precipitate overnight. Formation of the final product as a pale-yellow solid was observed, corresponding to the (*R*)-(4,4′-dibromo-[1,1′-binaphthalene]-2,2′-diyl)bis(diphenylphosphine) oxide product (**2**), which was isolated by filtration and washed with cold acetone several times, resulting in 72% yield. All the experimental data obtained for the characterization of **2** are consistent with the data found in the literature.^23^

^1^H-NMR (300 MHz, CDCl_3_): δ 8.21 (d, *J* = 8.4 Hz, 2H), 7.73 (s, 1H), 7.70–7.63 (m, 5H), 7.49–7.23 (m, 18H), 6.89–6.78 (m, 4H). ^31^P-NMR (400 MHz, CDCl_3_): δ 27.29; MALDI-TOF-MS: 813.0 (M + H)^+^, 835.0 (M + Na)^+^; Elemental analysis: theorical (C_44_H_30_Br_2_O_2_P_2_·4 H_2_O) % C 59.75, % H 4.33; exp.: % C 57.4, % H 3.6.

### 2.3 Synthesis of (*R*)-4,4′-(2,2′-Bis(diphenylphosphoryl)-[1,1′-binaphthalene]-4,4′-diyl)dibenzaldehyde (**3**)

In a 50 mL Schlenk tube with a magnetic stirrer and (*R*)-(4,4′-dibromo-[1,1′-binaphthalene]-2,2′-diyl)bis(diphenylphosphine) oxide (**2**) (770 mg, 0.95 mmol), (4-formylphenyl)boronic acid (298 mg, 1.99 mmol), potassium carbonate (420 mg, 3.03 mmol), and tetrakis(triphenylphosphine)-palladium (0) (165 mg, 0.14 mmol) were added. Then, the tube was sealed with a septum and three vacuum-Ar cycles were carried out before the addition of a purged tetrahydrofuran:water mixture (5:1, 12 mL) *via* syringe. Then, the reaction was heated to 80 °C under an Ar atmosphere and stirred for 72 h. When the reaction was completed, THF was removed under reduced pressure. The crude mixture was extracted with DCM, the organic layers were combined and dried over Na_2_SO_4_, and the solvent was removed under reduced pressure. The solid mixture obtained was crushed and washed with acetone, obtaining the final product as a pale-yellow solid in 80% yield. All the experimental data obtained for the characterization of **3** is consistent with a previous report.^24^

^1^H-NMR (300 MHz, CDCl_3_): δ 10.11 (s, 2H, -CHO), 8.00 (d, *J* = 8.0 Hz, 4H), 7.85 (d, *J* = 8.4 Hz, 2H), 7.73–7.63 (m, 8H), 7.49–7.34 (m, 12H), 7.29–7.20 (m, 8H), 7.05 (d, *J* = 8.5 Hz, 2H), 6.98–6.92 (m, 2H); ^31^P-NMR (400 MHz, CDCl_3_): δ 29.07 ppm; MALDI-TOF-MS: 863.3 (M + H)^+^, 885.3 (M + Na)^+^; Elemental analysis: theorical (C_58_H_40_O_4_P_2_ ·1.5 H_2_O): % C 78.28, % H 4.87; exp.: % C 78.1, % H 4.9.

### 2.4 Synthesis of the Imine-Based Chiral Organic Material (COM-Imine)

In a 19 mL vial, (*R*)-4,4′-(2,2′-bis(diphenylphosphoryl)-[1,1′-binaphthalene]-4,4′-diyl)dibenzaldehyde (**3**) (77 mg, 0.09 mmol), tris(4-aminophenyl)benzene (22 mg, 0.06 mmol), and scandium triflate (10 mg, 0.02 mmol) were dissolved in a MeOH:CHCl_3_ mixture (1:1, 10 mL). Then, the mixture was sonicated for 30 min (20 kHz), observing the formation of a yellow solid. The mixture was filtrated and washed sequentially with MeOH, DCM, and THF. Finally, the material was crushed and dried in vacuum overnight, obtaining 50 mg of **COM-Imine** material (53% yield) as an intense yellow solid.

### 2.5 Post-Synthetical Reduction of **COM-Imine** to Amine-Based Chiral Organic Material (**COM-Amine**)

In a 100 mL round-bottom flask, 270 mg of **COM-Imine** material was suspended in 45 mL of MeOH by sonication and the suspension was cooled down to 0 °C using an ice bath under stirring. Then, 2.87 g of NaBH_4_ was added portion-wise over the suspension. After the completion of the addition, the reaction was let overnight to be completed, at room temperature. The mixture was then filtrated and washed sequentially with distilled water, MeOH, and DCM. The isolated solid was dried overnight under vacuum, in order to obtain the **COM-Amine** material as a pale-yellow solid (185 mg, 69% yield).

### 2.6 General Procedure for Catalytic Allylation of Aromatic Aldehydes with Allyltrichlorosilane

In a 19 mL vial equipped with a magnetic stirrer, the COM catalyst (40 mg), the corresponding aldehyde (0.47 mmol), NBu_4_I (207 mg, 0.56 mmol), and DIPEA (410 μL, 2.35 mmol) were added. Then, 1 mL of DCM and allyltrichlorosilane (100 μL, 0.69 mmol) were sequentially added over the mixture and the reaction was stirred for 4 h at room temperature. After that time, 1 mL of a 10% w/w NaOH aqueous solution was added to quench the reaction and the internal standard 1,3,5-trimethoxybenzene (13 mg, 0.077 mmol) was added to the mixture. The crude was then filtrated using a syringe and a cellulose filter (0.2 μm ø), in order to remove any particle of the catalyst. Then, the liquid phase was extracted with AcOEt (3 × 10 mL). The organic layers were combined and washed sequentially with 5% w/w aqueous HCl solution (15 mL), saturated NaHCO_3_ aqueous solution (20 mL), and brine (20 mL). The organic phase was then dried over Na_2_SO_4_ and filtered, and AcOEt was removed under reduced pressure to afford the mixture, which would be directly analyzed by ^1^H-NMR and chiral chromatography.

## 3 Results and Discussion

### 3.1 Synthesis and Characterization of the Materials

**Scheme 2:**
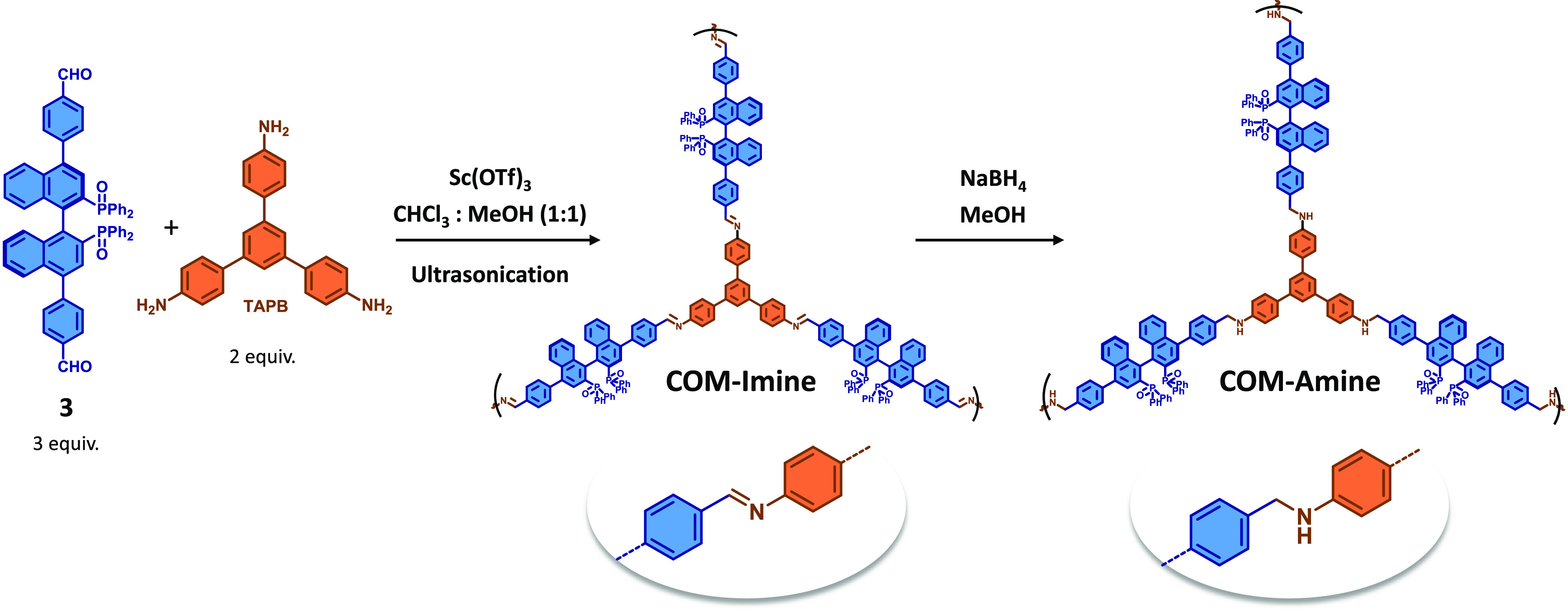
Synthetic Strategy for the Obtention of the Chiral Organic Material Based on Imine Condensation (**COM-Imine**) and Further Reduction to Afford the New Amine-Based Chiral Organic Material (**COM-Amine**)

First, we synthetized the building unit containing the atropoisomeric chiral BINAPO moiety according to a recent reported methodology.^24^ For this purpose, starting from the enantiomerically pure commercial (*R*)-BINAP, we performed consecutive oxidation, bromination, and Suzuki coupling synthetic steps to afford compound **3** (see Scheme 1). This building unit contains two benzaldehyde fragments located at the axial positions of the binaphthyl moiety, which enables the assembly of a COM through the imine condensation with the 1,3,5-tris(4-aminophenyl)benzene (**TAPB**) building block (Scheme 2). Methodologies commonly used in the synthesis of imine-based COFs, such as solvothermal procedures or room temperature synthesis in the presence of Brønsted or Lewis acids, did not afford significant yields of material (see Supporting Information, Table S1). Thus, we used more energetic reaction conditions inspired by recent reports using sonochemical procedures.^25^ Ultrasonication of the mixture of (*R*)-BINAPO-(PhCHO)_2_ (**3**) and **TAPB** building blocks in 1:1 MeOH:CHCl_3_ assisted by a Lewis acid catalyst (scandium(III) triflate) resulted in the obtention of an imine-based chiral organic material (**COM-Imine**), as a light-yellow solid. The need to make adequate the stability of the material to its final application as a heterogenous catalyst led us to perform the reductive post-functionalization of **COM-Imine**. Thus, we obtained the amine-linked analogous chiral organic material (**COM-Amine**), by reaction with NaBH_4_ following a procedure for the heterogeneous reduction of imines reported in literature.^26^

**Figure 2:**
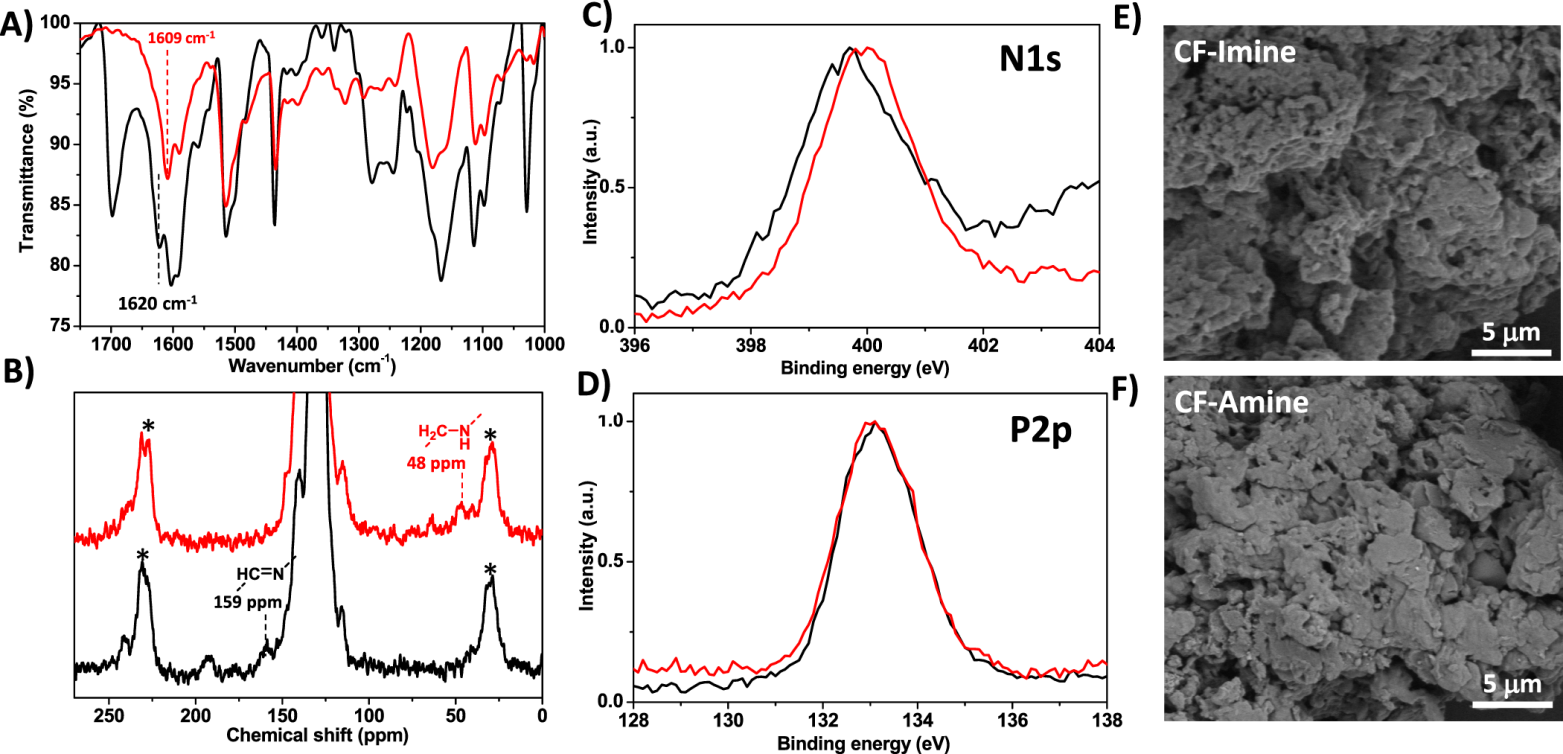
(A) FTIR spectra of **COM-Imine** (black) and **COM-Amine** (red) materials. (B) Solid-state CP-MAS ^13^C-NMR of **COM-Imine** (black) and **COM-Amine** (red) materials. The side spinning bands are denoted as (*). (C) Nitrogen 1s region of X-ray photoelectron spectra of **COM-Imine** (black) and **COM-Amine** (red) materials. (D) Phosphorus 2p region of X-ray photoelectron spectra of **COM-Imine** (black) and **COM-Amine** (red) materials. (E) SEM image of **COM-Imine** and (F) SEM image of **COM-Amine**.

A first comparative analysis of these materials by FTIR spectroscopy (Figure 2A) revealed the nature of the linkages present in each material structure; the peak at 1621 cm^–1^ that corresponds to the −C=N– stretching vibration of imine moieties can easily be identified in the **COM-Imine** spectrum. In addition, vibrations in the range of 1280–1240 cm^–1^ correspond to C–C=N–C stretching modes. These vibrations, as well as that associated with the peripheral aldehydes (1698 cm^–1^), disappear in the **COM-Amine** spectrum. Overall, the comparative infrared spectroscopy study confirms the reduction of the imine bonds to amines, which agrees well with previously reported reductions of imine-based COFs into their amine analogues.^26,27^

Further evidence of the imine-to-amine transformation was provided by solid-state cross polarization magic angle spinning (CP-MAS) ^13^C-NMR spectra of COM materials (Figure 2B). The peak at 153 ppm in the **COM-Imine** spectrum corresponds to the iminic carbon.^28^ This signal is not present in the **COM-Amine** spectrum. Another proof of this post-functionalization is the new band located at 48 ppm that can be observed in the **COM-Amine** spectrum, corresponding to the new C–N bond.^27^ Also, in good agreement with FTIR data, the peripheral carbonyl groups observed at 193 ppm in the **COM-Imine** spectrum disappear after the reductive post-functionalization. It is worth mentioning that the low abundance of imine/amine carbons compared with aromatic carbon nuclei present in both materials accounts for their relative low intensity in the CP-MAS ^13^C-NMR spectra.

Additional information of the chemical composition of both materials was obtained by X-ray photoelectron spectroscopy (XPS) analysis. We found signals associated with C, N, and P atoms in both samples (Figure 2C,D and Supporting Information, Figure S29), with the main difference observed in the binding energy of the N atom (Figure 2C). In particular, the N 1s signal of the **COM-Imine** sample was centered at 399.7 eV^29^ and shifted to 400 eV when the iminic linkages were reduced to amine bonds in the **COM-Amine** material. In order to estimate the position expected for the amine N atom in **COM-Amine**, we measured the N 1s signal of the triamine **TAPB** building block. In this control experiment, we observed a signal centered at the same binding energy than for **COM-Amine** (see Supporting Information, Figure S30). Thus, the small shift of 0.3 eV toward higher binding energies from the **COM-Imine** to **COM-Amine** samples is attributed to the effective reduction of the imine linkages into amine bonds. These results are in good agreement with NMR and FTIR data. Furthermore, the P 2p XPS signal does not present any differences between the two materials, which confirms the preservation of the phosphine oxide groups after the reduction process (Figure 2D).^30^ Finally, C 1s signals from the **COM-Imine** and **COM-Amine** materials were analyzed and no significant differences were observed between both spectra (see Supporting Information, Figure S31). This observation is related to the high abundance of aromatic carbon atoms relative to the imine/amine carbons in both materials, which should have a minor contribution in the overall observed signal.

Scanning electron microscopy (SEM) allowed us to analyze the microstructure of **COM-Imine** and **COM-Amine** materials (Figure 2E,F). In both cases, a layered microstructure is observed with lateral dimensions over 1 μm, as expected for their bidimensional molecular design. Interestingly, although the chemical nature of the materials is affected by the reductive post-functionalization, the morphology of the materials remains unaltered.

Thermal stability of both materials was assessed by thermogravimetric analysis measurements. A weight loss of 2–3% is observed around 100 °C, indicating the ability of COM materials to interact with solvent guest molecules (see Supporting Information, Figures S21 and S22). Furthermore, the chemical structure of the materials remains unaltered at least up to 300 °C.

Despite our efforts to optimize the crystallinity and porosity, trying to make adequate the synthetic procedures, the high steric hindrance imposed by BINAPO moieties hampered the structural order in the final materials. In addition, the preservation of porosity in COM materials and their efficient stacking is disfavored by their large pore size (around 3.4 nm), which makes more probable a defective material’s growth. Consequently, X-ray diffraction measurements and N_2_ adsorption–desorption isotherms revealed their amorphous and non-porous nature (see Supporting Information, Figures S28, S23, and S24). However, CO_2_ adsorption analysis revealed a Langmuir surface area value of 254 m^2^/g for the COM-Amine material, which can be due to an enhanced affinity of this material to such polar adsorbate (Figure S25). In addition, to demonstrate the transfer of chirality from the building unit **3** to **COM-Amine**, we performed polarimetry measurements on a methanol suspension of this material. Interestingly, we observed a positive optical rotation that decreased over time due to deposition of the suspended solid. This behavior is in the same direction than the previously reported data for solutions of molecular (*R*)-BINAPO^31^ and also for our measurements on the building block **3**.

The common definition of COF includes the requirement of porosity and crystallinity regardless of chemical composition or functionality.^32^ Thus, similar chemical designs may or may not fit the definition of COF depending on their good crystalline packing. However, catalytic properties are not necessarily correlated with crystallinity and porosity.^33,34^ In this work, we present a novel design of a COF-type organocatalytic structure, referred to as chiral organic material, since it resulted in an amorphous and non-porous material. Nevertheless, catalytic activities and selectivities observed are satisfactory and compete with data reported for homogeneous systems.

### 3.2 Asymmetric Organocatalytic Activity: Allylation of Aromatic Aldehydes with Allyl Trichlorosilane

**Scheme 3:**
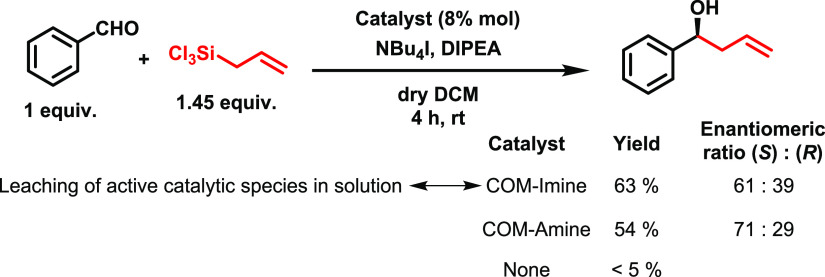
Model Reaction Explored to Initially Assess the Catalytic Performance of **COM-Imine** and **COM-Amine** Materials

The organocatalytic properties of **COM-Imine** and **COM-Amine** materials were initially assessed by testing the asymmetric allylation of benzaldehyde with allyl trichlorosilane as a model reaction under the same conditions previously described in the literature for the molecular BINAPO catalyst (Scheme 3). To our delight, the results obtained for this reaction using these materials as heterogeneous catalysts were comparable to data reported for homogeneous systems.^17^ However, the possibility of leaching of molecular active species into the reaction medium during the catalytic process in the presence of highly nucleophilic allylic trichlorosilane should be checked. For this purpose, after the first catalytic run, the reaction mixture was filtered to remove the heterogeneous COM catalysts. Then, additional amounts of benzaldehyde and trichlorosilane were added to the filtered crudes. The mixture was allowed to react for another catalytic run, and the progression of the reaction was followed by ^1^H-NMR. Generation of additional amounts of the product would be indicative of the undesired homogeneous nature of the catalytic activity. Indeed, when this experimental procedure was carried out using the **COM-Imine** material, we observed that the filtrated solution afforded an additional 0.21 mmol of alcohol product **4a**. However, if the **COM-Amine** material is used, the filtrated solution does not generate significant extra amounts of products. Thus, while leaching of catalytically active species was observed in the case of the **COM-Imine** material, this phenomenon was discarded for the **COM-Amine** catalyst (see the Supporting Information). These results confirmed that the **COM-Amine** material presents higher stability than **COM-Imine** under the reaction conditions while preserving the activity and enantioselectivity properties previously observed for the molecular BINAPO catalyst. In fact, both the allyltrichlorosilane and the aldehydes used may affect the integrity of the material. On the one hand, it is known that imines react in the presence of nucleophilic reagents, causing the disassembly of COFs based on this type of bond.^19,20^ Thus, highly nucleophilic silane derivatives can be responsible of disassembling of **COM-Imine**. On the other hand, since imines are a highly reversible bond, the presence of aldehyde groups can lead to the dynamic disassembly of imine-based materials.^35^

**Figure 3:**
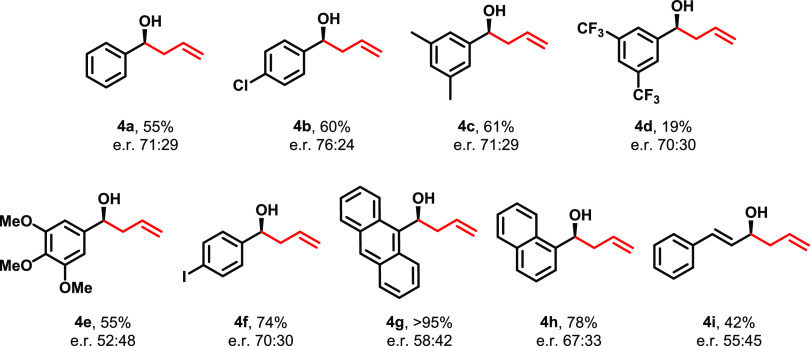
Scope of asymmetric allylation of aldehydes with allyltrichlorosilane catalyzed by **COM-Amine**. Yields obtained by ^1^H-NMR peak integration of the worked-up reaction crude using 1,3,5-trimethoxybenzene as the internal standard. The enantiomeric ratio was obtained by comparing and integrating UV–visible absorbance peaks after purifying the worked-up crude by SFC. e.r. = enantiomeric ratio.

We investigated the applicability of this heterogeneous system with a variety of aromatic aldehyde substrates with different substituents in their structure (Figure 3), obtaining the corresponding allylated alcohol derivatives in moderate-to-good yields. The absolute configuration of the product from the reaction of benzaldehyde was determined by polarimetry measurements, with the (*S*)-1-phenylbut-3-en-1-ol isomer (**4a**) being the major product (see the Supporting Information).^36^ Owing to similar structures of aromatic aldehyde precursors, we assumed that the absolute configuration of the products is preserved as that observed for benzaldehyde. The results obtained demonstrated the applicability of this system either for electron-withdrawing (**4d**) or for electron-donating groups (**4c**, **4e**) in the aromatic aldehyde substrates. The system also tolerates the presence of halogen substituents in the *para* position (**4b**, **4f**). Interestingly, although the naphthyl (**4h**) and anthracene (**4g**) substituents are highly sterically hindered, the yields obtained for these substrates are excellent.

**Figure 4:**
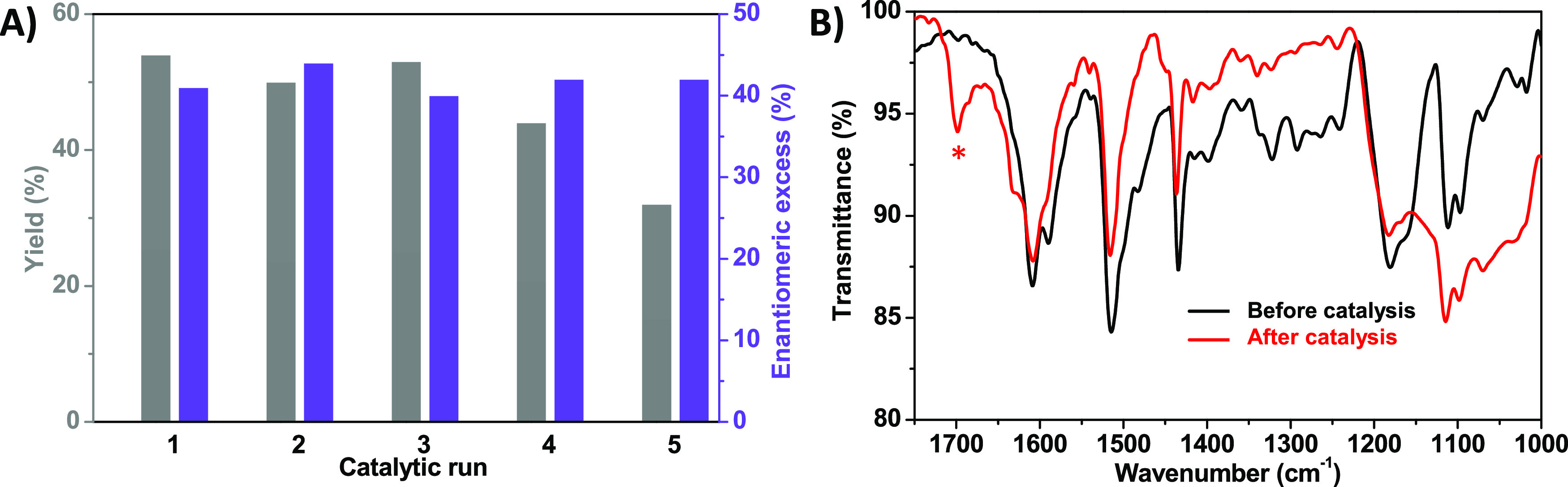
Recyclability of **COM-Amine** for the reaction of allyltrichlorosilane and benzaldehyde: (A) yields and enantiomeric excesses found for five consecutive catalytic runs. (B) FTIR spectra recorded before and after catalysis (* denotes the signal corresponding to residual benzaldehyde used as the catalytic substrate).

The final requirement for an asymmetric heterogeneous catalyst consists of its recyclability, while preserving similar yields and enantiomeric excesses after each catalytic run. To test this notion, we carried out the model reaction with the same catalyst sample for five catalytic runs. After each cycle, we separated the catalyst from the crude mixture by centrifugation; the catalyst was then washed with ethyl acetate and reused (see Supporting Information). As can be observed in Figure 4, the **COM-Amine** material was able to perform such reaction during at least five cycles, leading to similar yields and enantiomeric excesses in each run (Figure 4A). These results confirm not only the recyclability of the system, but also the reproducibility of our experimental setup, even though after the fourth and fifth cycles the yields slightly decreased, probably due to small losses of catalyst load during the separation process. Importantly, we found that enantioselectivity is always preserved regardless of the catalytic cycle.

Finally, we compared the corresponding FTIR spectra before and after catalysis (Figure 4B) to study the effect of these catalytic runs in the structure of the material. The main characteristic bands of **COM-Amine** remain after catalysis, proving the resistance of the material to the reaction conditions. However, we can observe that some new bands have appeared in the FTIR spectrum recorded after catalysis. Residues of the benzaldehyde reagent are noted by the aldehyde characteristic signal at 1700 cm^–1^ corresponding to stretching of C=O. This is ascribed to the ability of benzaldehyde to interact with the conjugated organic material.

## 4 Conclusions

A novel imine-based organic material that contains the chiral phosphine oxide BINAPO in its backbone (**COM-Imine**) has been obtained combining Lewis acid catalysis and sonochemistry. However, the low stability of imine bridges prevents the use of **COM-Imine** as a heterogeneous catalyst in asymmetric allylation of aromatic aldehydes. To overcome this drawback, reductive post-functionalization of imines into amines is demonstrated to be an efficient strategy to stabilize this organic framework. Thus, the amine-linked material containing BINAPO (**COM-Amine**) has been successfully used as a heterogeneous chiral organocatalyst showing activities and selectivities comparable to its molecular analogue but showing a good degree of recyclability. Overall, this work expands the application of reticular designs of organic materials for challenging asymmetric organocatalytic transformations under demanding reaction conditions.

## Supporting Information Available

General materials and methods; optimization of COM formation conditions; asymmetric allylation of aromatic aldehydes; model reaction’s control experiment with molecular catalyst binaphtylphosphine oxide (BINAPO); catalytic structure of the materials, number of active catalytic sites, and TON; leaching experiments; recyclability experiments; ^1^H-NMR and ^31^P-NMR spectra of the building unit’s precursors (**1** and **2**) and building unit (**3**) (CDCl_3_, 300 MHz for ^1^H and 500 MHz for ^31^P); MALDI-TOF-mass spectra of the building unit’s precursors (**1** and **2**) and building unit (**3**); FT-IR spectra of building unit **3** and TAPB; thermogravimetric analyses of COM-Imine and COM-Amine materials; N_2_ adsorption–desorption isotherms of COM-Imine and COM-Amine materials; ^31^P-NMR spectra of COM-Imine and COM-Amine materials; X-ray diffraction patterns from COM-Imine and COM-Amine materials; X-ray photoelectron spectra of COM-Imine and COM-Amine; FT-IR spectrum of COM-Imine and COM-Amine materials; characterization of the products of the aldol addition reactions (PDF)

## References

[ref1] García Mancheño O.; Waser M. Recent Developments and Trends in Asymmetric Organocatalysis. Eur. J. Org. Chem. 2023, 26, e202200950 10.1002/ejoc.202200950. PMC10091998 37065706

[ref2] Fulgheri T.; Della Penna F.; Baschieri A.; Carlone A. Advancements in the Recycling of Organocatalysts: From Classical to Alternative Approaches. Curr. Opin. Green Sustainable Chem. 2020, 25, 100387 10.1016/j.cogsc.2020.100387.

[ref3] Susam Z. D.; Tanyeli C. Recyclable Organocatalysts in Asymmetric Synthesis. Asian J. Org. Chem. 2021, 10, 1251–1266. 10.1002/ajoc.202100165.

[ref4] Valverde-González A.; Fernández-Seriñan P.; Matarín Á.; Arnanz A.; Sánchez F.; Iglesias M. Porous Aromatic Frameworks Containing Binaphthyl-dihydroazepine Units (cBAPAFs) as Catalytic Supports for Asymmetric Reactions. J. Catal. 2022, 413, 434–442. 10.1016/j.jcat.2022.06.034.

[ref5] Yu S.-C.; Cheng L.; Liu L. Asymmetric Organocatalysis with Chiral Covalent Organic Frameworks. Org. Mater. 2021, 03, 245–253. 10.1055/a-1400-5581.

[ref6] Debruyne M.; Van Speybroeck V.; Van Der Voort P.; Stevens C. V. Porous Organic Polymers as Metal Free Heterogeneous Organocatalysts. Green Chem. 2021, 23, 7361–7434. 10.1039/D1GC02319E.

[ref7] Daliran S.; Oveisi A. R.; Peng Y.; López-Magano A.; Khajeh M.; Mas-Ballesté R.; Alemán J.; Luque R.; Garcia H. Metal-Organic Framework (MOF)-, Covalent-Organic Framework (COF)-, and Porous-Organic Polymers (POP)-Catalyzed Selective C-H Bond Activation and Functionalization Reactions. Chem. Soc. Rev. 2022, 51, 7810–7882. 10.1039/D1CS00976A. 35938695

[ref8] López-Magano A.; Daliran S.; Oveisi A. R.; Mas-Ballesté R.; Dhakshinamoorthy A.; Alemán J.; Garcia H.; Luque R. Recent Advances in the Use of Covalent Organic Frameworks as Heterogeneous Photocatalysts in Organic Synthesis. Adv. Mater. 2023, 220947 10.1002/adma.202209475. 36563668

[ref9] Tran Q. N.; Lee H. J.; Tran N. Covalent Organic Frameworks: From Structures to Applications. Polymer 2023, 15, 1279 10.3390/polym15051279. PMC10007052 36904520

[ref10] Wang X.; Han X.; Zhang J.; Wu X.; Liu Y.; Cui Y. Homochiral 2D Porous Covalent Organic Frameworks for Heterogeneous Asymmetric Catalysis. J. Am. Chem. Soc. 2016, 138, 12332–12335. 10.1021/jacs.6b07714. 27618953

[ref11] Zhang J.; Han X.; Wu X.; Liu Y.; Cui Y. Chiral DHIP- and Pyrrolidine-Based Covalent Organic Frameworks for Asymmetric Catalysis. ACS Sustainable Chem. Eng. 2019, 7, 5065–5071. 10.1021/acssuschemeng.8b05887.

[ref12] Xu H.; Chen X.; Gao J.; Lin J.; Addicoat M.; Irle S.; Jiang D. Catalytic Covalent Organic Frameworks via Pore Surface Engineering. Chem. Commun. 2014, 50, 1292–1294. 10.1039/C3CC48813F. 24352109

[ref13] Han X.; Zhang J.; Huang J.; Wu X.; Yuan D.; Liu Y.; Cui Y. Chiral Induction in Covalent Organic Frameworks. Nat. Commun. 2018, 9, 1294 10.1038/s41467-018-03689-9. 29615606 PMC5882852

[ref14] Kan X.; Wang J. C.; Chen Z.; Du J. Q.; Kan J. L.; Li W. Y.; Dong Y. B. Synthesis of Metal-Free Chiral Covalent Organic Framework for Visible-Light-Mediated Enantioselective Photooxidation in Water. J. Am. Chem. Soc. 2022, 144, 6681–6686. 10.1021/jacs.2c01186. 35394764

[ref15] Ma H.-C.; Sun Y.-N.; Chen G.-J.; Dong Y.-B. A BINOL-Phosphoric Acid and Metalloporphyrin Derived Chiral Covalent Organic Framework for Enantioselective α-Benzylation of Aldehydes. Chem. Sci. 2022, 13, 1906–1911. 10.1039/D1SC06045G. 35308838 PMC8848806

[ref16] Ayad T.; Gernet A.; Pirat J.-L.; Virieux D. Enantioselective Reactions Catalyzed by Phosphine Oxides. Tetrahedron 2019, 75, 4385–4418. 10.1016/j.tet.2019.06.042.

[ref17] Kotani S.; Hashimoto S.; Nakajima M. Chiral Phosphine Oxide BINAPO as a Lewis Base Catalyst for Asymmetric Allylation and Aldol Reaction of Trichlorosilyl Compounds. Tetrahedron 2007, 63, 3122–3132. 10.1016/j.tet.2007.02.014.

[ref18] Qian C.; Feng L.; Teo W. L.; Liu J.; Zhou W.; Wang D.; Zhao Y. Imine and Imine-Derived Linkages in two-Dimensional Covalent Organic Frameworks. Nat. Rev. Chem. 2022, 6, 881–898. 10.1038/s41570-022-00437-y. 37117702

[ref19] Jiménez-Almarza A.; López-Magano A.; Mas-Ballesté R.; Alemán J. Tuning the Activity–Stability Balance of Photocatalytic Organic Materials for Oxidative Coupling Reactions. ACS Appl. Mater. Interfaces 2022, 14, 16258–16268. 10.1021/acsami.2c01646. 35348315 PMC9011354

[ref20] Jin E.; Geng K.; Lee K. H.; Jiang W.; Li J.; Jiang Q.; Irle S.; Jiang D. Topology-Templated Synthesis of Crystalline Porous Covalent Organic Frameworks. Angew. Chem., Int. Ed. 2020, 59, 12162–12169. 10.1002/anie.202004728. 32329936

[ref21] Cusin L.; Peng H.; Ciesielski A.; Samorì P. Chemical Conversion and Locking of the Imine Linkage: Enhancing the Functionality of Covalent Organic Frameworks. Angew. Chem., Int. Ed. 2021, 60, 14236–14250. 10.1002/anie.202016667. 33491860

[ref22] Grunenberg L.; Savasci G.; Terban M. W.; Duppel V.; Moudrakovski I.; Etter M.; Dinnebier R. E.; Ochsenfeld C.; Lotsch B. V. Amine-Linked Covalent Organic Frameworks as a Platform for Postsynthetic Structure Interconversion and Pore-Wall Modification. J. Am. Chem. Soc. 2021, 143, 3430–3438. 10.1021/jacs.0c12249. 33626275 PMC7953377

[ref23] Alamé M.; Meille V.; De Bellefon C.; Jahjah M.; Pellet-Rostaing S.; Berthod M.; Lemaire M. Highly Regioselective Bromination of BINAP in [Hmim]PF6 Ionic Liquid. Synth. Commun. 2007, 38, 141–147. 10.1080/00397910701651342.

[ref24] Du C.; Zhu X.; Yang C.; Liu M. Stacked Reticular Frame Boosted Circularly Polarized Luminescence of Chiral Covalent Organic Frameworks. Angew. Chem., Int. Ed. 2022, 61, e202113979 10.1002/anie.202113979. 34693602

[ref25] Zhao W.; Yan P.; Yang H.; Bahri M.; James A. M.; Chen H.; Liu L.; Li B.; Pang Z.; Clowes R.; Browning N. D.; Ward J. W.; Wu Y.; Cooper A. I. Using Sound to Synthesize Covalent Organic Frameworks in Water. Nat. Synth. 2022, 1, 87–95. 10.1038/s44160-021-00005-0.

[ref26] Liu H.; Chu J.; Yin Z.; Cai X.; Zhuang L.; Deng H. Covalent Organic Frameworks Linked by Amine Bonding for Concerted Electrochemical Reduction of CO2. Chem 2018, 4, 1696–1709. 10.1016/j.chempr.2018.05.003.

[ref27] Zhang M.; Li Y.; Yuan W.; Guo X.; Bai C.; Zou Y.; Long H.; Qi Y.; Li S.; Tao G.; Xia C.; Ma L. Construction of Flexible Amine-linked Covalent Organic Frameworks by Catalysis and Reduction of Formic Acid via the Eschweiler–Clarke Reaction. Angew. Chem., Int. Ed. 2021, 60, 12396–12405. 10.1002/anie.202102373. 33682274

[ref28] Jiménez-Almarza A.; López-Magano A.; Marzo L.; Cabrera S.; Mas-Ballesté R.; Alemán J. Imine-Based Covalent Organic Frameworks as Photocatalysts for Metal Free Oxidation Processes under Visible Light Conditions. ChemCatChem 2019, 11, 4916–4922. 10.1002/cctc.201901061.

[ref29] Lu H.; Ning F.; Jin R.; Teng C.; Wang Y.; Xi K.; Zhou D.; Xue G. Two-Dimensional Covalent Organic Frameworks with Enhanced Aluminum Storage Properties. ChemSusChem 2020, 13, 3447–3454. 10.1002/cssc.202000883. 32368825

[ref30] Biagiotti G.; Langè V.; Ligi C.; Caporali S.; Muniz-Miranda M.; Flis A.; Pietrusiewicz K. M.; Ghini G.; Brandi A.; Cicchi S. Nanostructured Carbon Materials Decorated with Organophosphorus Moieties: Synthesis and Application. Beilstein J. Nanotechnol. 2017, 8, 485–493. 10.3762/bjnano.8.52. 28326239 PMC5331327

[ref31] Berthod M.; Mignani G.; Lemaire M. New Perfluoroalkylated BINAP Usable as a Ligand in Homogeneous and Supercritical Carbon Dioxide Asymmetric Hydrogenation. Tetrahedron: Asymmetry 2004, 15, 1121–1126. 10.1016/j.tetasy.2004.02.004.

[ref32] Lyle S. J.; Waller P. J.; Yaghi O. M. Covalent Organic Frameworks: Organic Chemistry Extended into Two and Three Dimensions. Trends Chem. 2019, 1, 172–184. 10.1016/j.trechm.2019.03.001.

[ref33] López-Magano A.; Salaverri N.; Marzo L.; Mas-Ballesté R.; Alemán J. Synergistic Combination of Triazine and Phenanthroline Moieties in a Covalent Triazine Framework Tailored for Heterogeneous Photocatalytic Metal-Free C-Br and C-Cl Activation. Appl. Catal., B 2022, 317, 121791 10.1016/j.apcatb.2022.121791.

[ref34] López-Magano A.; Ortín-Rubio B.; Imaz I.; Maspoch D.; Alemán J.; Mas-Ballesté R. Photoredox Heterobimetallic Dual Catalysis Using Engineered Covalent Organic Frameworks. ACS Catal. 2021, 11, 12344–12354. 10.1021/acscatal.1c03634. 34900388 PMC8650013

[ref35] Vitaku E.; Dichtel W. R. Synthesis of 2D Imine-Linked Covalent Organic Frameworks through Formal Transimination Reactions. J. Am. Chem. Soc. 2017, 139, 12911–12914. 10.1021/jacs.7b06913. 28853570

[ref36] Minowa N.; Mukaiyama T. Asymmetric Allylation with a New Chiral Allylating Agent Prepared from Tin(II) Triflate, Chiral Diamine, and Allylaluminum. Bull. Chem. Soc. Jpn. 1987, 60, 3697–3704. 10.1246/bcsj.60.3697.

