# Supplementary material for: Stabilized Chiral Organic Material Containing BINAP Oxide Units as a Heterogeneous Asymmetric Organocatalyst for Allylation of Aldehydes
Source: ACS Appl Mater Interfaces. 2023 Jun 12;15(25):30212–9. doi: 10.1021/acsami.3c04430 (PMC10316330; doi:10.1021/acsami.3c04430)
Supplement: Supplementary file 1 — am3c04430_si_001.pdf [file am3c04430_si_001.pdf]

Supporting Information

Stabilized chiral organic material containing BINAP oxide units as heterogeneous asymmetric organocatalyst for allylation of aldehydes.

Miguel Sánchez-Fuente,[a] *Alberto López-Magano,*[a] *Alicia Moya* [a,*] *and Rubén Mas-Ballesté* [a,b,*].

a. Department of Inorganic Chemistry (Module 7), Facultad de Ciencias, Universidad Autónoma de Madrid, 28049 Madrid, Spain.

b. Institute for Advanced Research in Chemical Sciences (IAdChem), Universidad Autónoma de Madrid, 28049 Madrid, Spain.

E-mail: alicia.moya@uam.es; ruben.mas@uam.es

Table of contents

1. General materials and methods 3
2. Optimization of COM formation conditions 4
3. Asymmetric allylation of aromatic aldehydes....5
4. Model reaction´s control experiment with molecular catalyst Binaphtylphosphine oxide (BINAPO) .6
5. Catalytic structure of the materials, number of active catalytic sites and TON 6
6. Leaching experiments 7
7. Recyclability experiments 10
8. ^{1}H-NMR and ^{31}P-NMR spectra of building unit´s precursors (1 and 2) and building unit (3) ($CDCl_3$, 300 MHz for ^{1}H and 500 MHz for ^{31}P) 14
9. MALDI-TOF-mass spectra of building unit´s precursors (1 and 2) and building unit (3) 17
10. FT-IR spectra of the building unit **3** and TAPB 19
11. Thermogravimetric analyses of COM-Imine and COM-Amine materials 20
12. N_2 adsorption-desorption isotherms of COM-Imine and COM-Amine materials 21
13. ^{31}P-NMR spectra of COM-Imine and COM-Amine materials 23
14. X-ray diffraction patterns from COM-Imine and COM-Amine materials....24
15. X-ray photoelectron spectra of COM-Imine and COM-Amine 24
16. FT-IR spectrum of COM-Imine and COM-Amine materials 26
17. Characterization of the products of the aldol addition reactions. 27
18. References 36

1. General materials and methods

All reagents and solvents were commercially available (*Sigma Aldrich*, *Fluorochem* or *BLD Pharm*).

Nuclear magnetic resonance (NMR) spectra were acquired on a *Bruker AV-300* spectrometer (*Bruker Corporation*, Billerica, MA, USA), running at 300 MHz for ^1H and at 75 MHz for ^{13}C. For the ^{31}P-NMR analyses a *Bruker AV-400* was employed. Chemical shifts (δ) are reported in ppm relative to residual solvent signals ($CDCl_3$: 7.26 ppm for ^1H-NMR, 77.0 ppm for ^{13}C-NMR). ^{13}C solid-state nuclear magnetic resonance was acquired on a *Bruker AV-400* spectrometer coupled to a multinuclear probe (^{15}N-^{31}P) CPMAS with triple channel (BL4 X/Y/^1H) for a 4 mm rotor at room temperature, using 1k scans and 12 kHz of turning speed. The ^1H excitation pulse used is $\pi/2 \times 2.75$ µs and the contact pulse is 3 ms.

X-ray photoelectron spectroscopy (XPS) measurements were carried out under ultra-high vacuum conditions (10^{-10} mbar) using a *SPECS GmbH* electron spectroscopy system provided with a *PHOIBOS 150 9MCD* analyzer, and a double anode X-ray source (Al/Mg).

Elemental chemical analyses were obtained in an elemental analyzer *LECO CHNS-932* model number 601-800-500.

FT-IR spectra were recorded in a *Perkin-Elmer 283* equipped with ATR MIRacle Single Reflection Horizontal.

MALDI-TOF mass spectrometry analyses were obtained employing a *Bruker ULTRAFLEX III* set up on a positive ion detection mode and using a DCTB and a NaI-doped DCTB as matrixes for the samples.

Powder X-ray diffraction: PXRD patterns were obtained in a *X'Pert PRO* diffractometer θ/2θ geometry from *Panalytical* equipped with a *Johansson* monochromator for ʎ Kα, a *X'Celerator* fast detector in an alumina holder. The θ/2θ swept was performed from 1° to 45° with an angular increase of 0.0167°/100 s.

Volumetric N_2 adsorption-desorption isotherms were collected at 77 K (N_2) using an *ASAP 2020* equipment from *Micromeritics*. Temperature was controlled by using a liquid nitrogen bath.

Volumetric CO_2 adsorption isotherms were collected at 273 K using an *ASAP 2020* equipment from *Micromeritics*.

Scanning electron microscopy (SEM) images were carried out on a *Hitachi S-3000N* electron microscope with a coupled ESED detector and an analyzer from energy dispersive X-ray from *Oxford Instruments, INCAx-sight* model. The images were obtained in vacuum after being metallized in a *Sputter Quórum Q150T-S* with gold coating.

Thermogravimetric analyses (TGA) were performed on a *TGA Q500 Thermobalance* from *TA Instruments*, heating the sample from 25 ^oC to 900 ^oC at 10 ^oC/min under air atmosphere.

Ultrasonication was carried out using an *Homogeneizator CY-500* (*Optic Ivymen Systems™, COMECTA®*) of 20 KHz, set up to 35% amplitude and at 24 °C.

Optical rotations were measured on an *Anton Paar NCP 100* polarimeter at room temperature and $[\alpha]_D^{23}$ values are given in deg·mL·g^{-1}·dm^{-1}. The experimental value obtained from the measurements of a solution of the isolated 1-phenylbut-3-en-1-ol product in benzene, (c 1.2 g/100 mL) was $[\alpha]_D^{23}$ -17.2. According to the literature, this result can be attributed to an excess of the (*S*)-1-phenylbut-3-en-1-ol isomer, which is considered as the major product of the reaction.[1]

Supercritical fluid chromatography for enantiomers´ separation: the chiral separation of the enantiomers in the crude mixtures was performed on an SFC Agilent Technologies 1260 Infinity Series instrument equipped with a UV-VIS detector employing a *Daicel Chiralpak* and columns IA, IB, ID and IG columns as chiral stationary phases, on the conditions specified below for each product.

2. Optimization of COM formation conditions

Table S1: Optimization of the synthetic conditions for COM-Imine

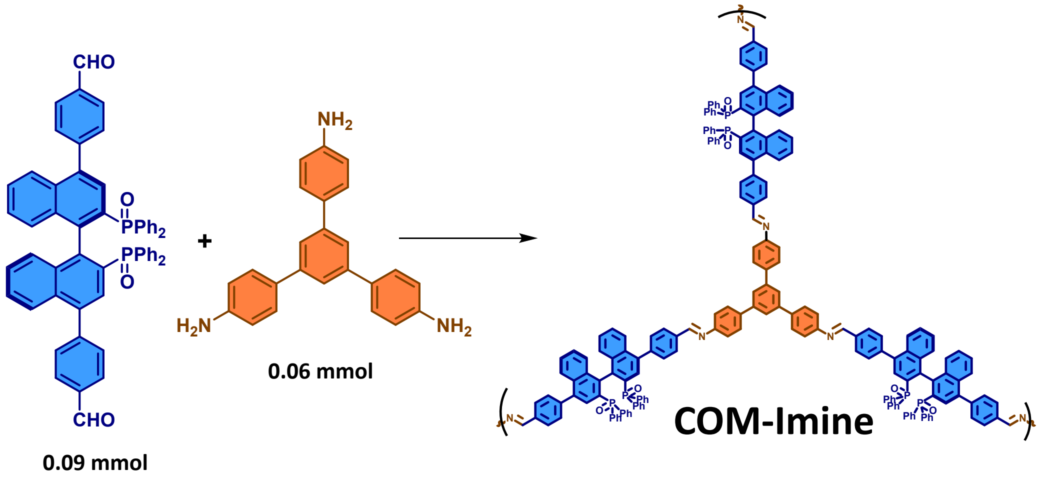

Solvent	Additive	Sonication	COM-Imine Formation
$CHCl_3$: MeOH (1:1) 10.0 mL	Acetic acid (6M) 0.6 mL	-	N.R.
$CHCl_3$: MeOH (1:1) 10.0 mL	Acetic acid (6M) 0.6 mL	20 KHz, 35 %, 30 min	< 20 %
$CHCl_3$ 10.0 mL	$Sc(OTf)_3$ (0.004 mmol)	20 KHz, 35 %, 30 min	< 20 %
$CHCl_3$: MeOH (1:1) 10.0 mL	$Sc(OTf)_3$ (0.004 mmol)	20 KHz, 35 %, 30 min	< 20 %
$CHCl_3$: MeOH (1:1) 10.0 mL	$Sc(OTf)_3$ (0.02 mmol)	-	< 20 %
1,2-Dichlorobenzene 10.0 mL	$Sc(OTf)_3$ (0.02 mmol)	20 KHz, 35 %, 30 min	< 20 %

$CHCl_3$: MeOH (1:1) 10.0 mL	$Sc(OTf)_3$ (0.02 mmol)	20 KHz, 35 %, 30 min	53 %
$CHCl_3$: MeOH (1:1) 20.0 mL	$Sc(OTf)_3$ (0.02 mmol)	20 KHz, 35 %, 30 min	< 20 %

3. Asymmetric allylation of aromatic aldehydes.

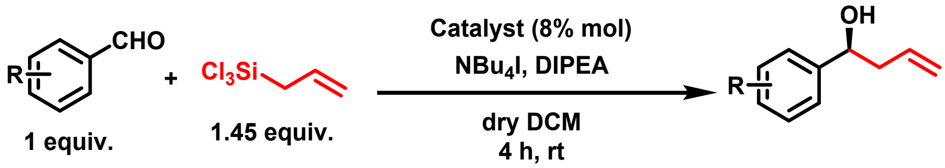

Scheme S1: Model asymmetric allylation reaction

In a 19 mL vial equipped with a magnetic stirrer, COM catalyst (40 mg), the corresponding aldehyde (0.47 mmol), NBu_4I (207 mg, 0.56 mmol) and DIPEA (410 µL, 2.35 mmol) were added. Then, 1 mL of DCM was added to the mixture. Then, allyltrichlorosilane (100 µL, 0.69 mmol) was added over the mixture, and the reaction was stirred for 4 h at room temperature. After that time, 1 mL of a 10% w/w NaOH aq. solution was added to quench the reaction, and the internal standard TMB (13 mg, 0.077 mmol) was added to the mixture. The crude was then filtrated using a syringe and a cellulose filter (0.2 µm ø), in order to remove any particle of the catalyst. Then, the liquid phase was extracted with AcOEt (3 x 10 mL). The organic layers were combined and washed sequentially with 5% w/w aq. HCl solution (15 mL), sat. $NaHCO_3$ aq. solution (20 mL) and brine (20 mL). The organic phase was then dried over Na_2SO_4, filtered and the AcOEt was removed under reduced pressure to afford the mixture. The yields of the reactions were calculated by ^{1}H-NMR peak integration of the worked-up crudes from the reactions, by adding 1,3,5-tri(methoxy)benzene (TMB, 13 mg, 0.08 mmol) to the mixture before the work-up, and comparing the signal of TMB at 6.10 ppm (s, 3H) and characteristic signals of the products, commonly the best isolated signal for 1H of each spectrum. As a control experiment, we tested the model aldol reaction with benzaldehyde in absence of catalyst, observing no product in the crude mixture.

4. Model reaction´s control experiment with molecular catalyst Binaphtylphosphine oxide (BINAPO)

The standard procedure for the model asymmetric allylation reaction was performed as depicted in *Scheme 1* (based on a method reported in literature[2]), using the (*R*)-BINAP Oxide (30.8 mg, 0.047 mmol, 10% mol) as catalyst instead of COM-catalysts, and the reaction crude was analyzed as usual. The product **4a** was obtained in 90% yield and 36% *ee*.

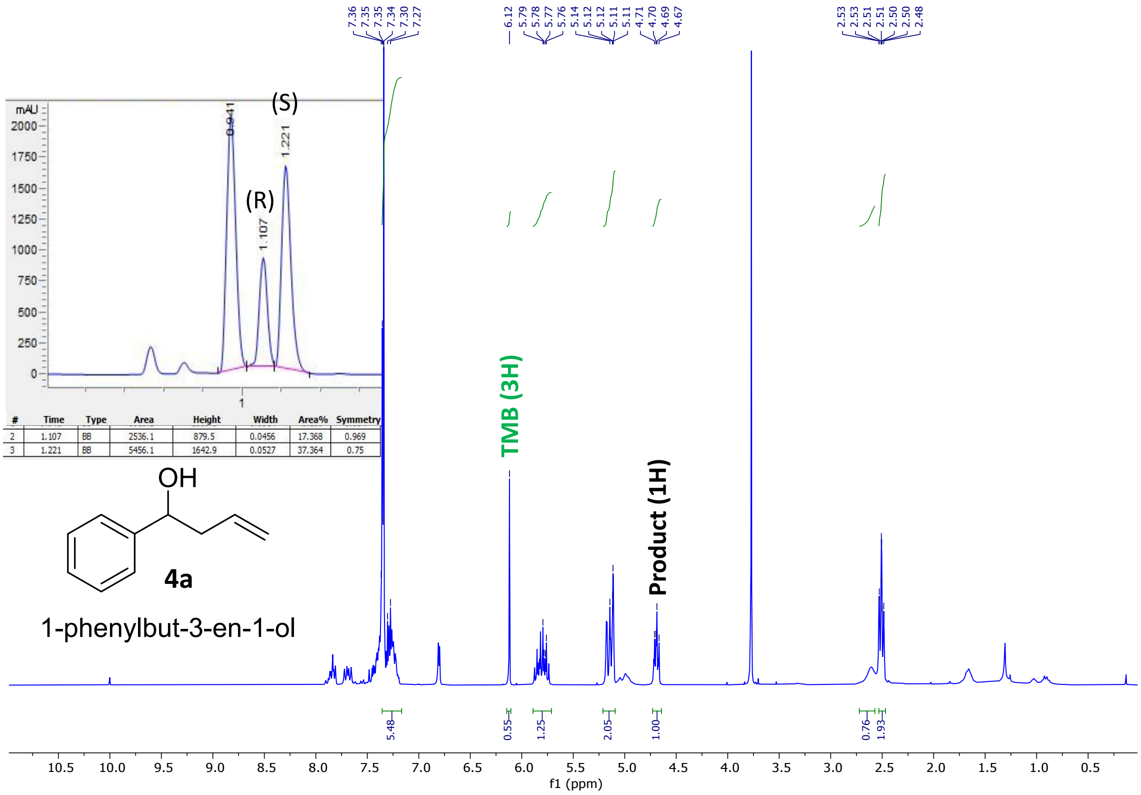

Figure S1: ^{1}H-NMR ($CDCl_3$, 300MHz) and SFC-Chromatogram of the crude from the homogeneous system reaction, containing product ***4a.*** *SFC Conditions: IB-Column, isocratic 5 % MeOH in supercritical CO_2, 10 min.*

5. Catalytic structure of the materials, number of active catalytic sites and TON

Table S2: calculated TON for the COM-Imine and COM-Amine catalysts in the model reaction experiments (0.47 mmol of benzaldehyde as limiting reagent and 40 mg of COM catalyst). Considering the molecular weight of building blocks, the reaction condensation of imine and the hydrogenation step of imine to amine, we calculated that the concentration of BINAPO sites in both COM-Imine and COM-Amine materials

is approximately $9.4 \cdot 10^{-4}$ *mmol of BINAPO per mg of both COM-Imine and COM-Amine.*

Catalyst	Model reaction Yield (%)	TON	Active BINAPO sites catalytic loading (mol %)
COM-Imine	57	7.13	8
COM-Amine	54	6.75	8

6. Leaching experiments

To evaluate the leaching of active catalytic species, the model aldol reaction with benzaldehyde was carried out as described in the Experimental section with both COM-Imine and COM-Amine materials. After the reaction was completed, avoiding the quenching step of the reaction with 1 mL of 10% w/w NaOH solution, the internal standard TMB (13 mg, 0.08 mmol) was added to the crude mixtures. Then, they were filtered with a syringe and a cellulose filter (0.2 µm pore) in order to remove any particle of COM catalysts. These crudes were transferred to another 19 mL glass vial and reloaded with the same amounts of benzaldehyde (extra 0.47 mmol) and trichlorosilane (extra 0.69 mmol). The new mixtures were let to react for another 4 h. The new crudes were worked-up as usual and analyzed by ^{1}H-NMR. We compared the relation of the signals of the internal standard (TMB, 6.10 ppm, s (3H)) and the product (4.75ppm, dd (1H)) from the crudes obtained after 2 reaction runs with those the relations of signals present in the crudes obtained from the first reaction run. This relation of signals was the same in the case of the COM-Amine for the two experiments, revealing that the second run (after filtrating the crude from the first run) did not afford any additional yield. However, in the case of COM-Imine, we observed that the filtrated solution from the COM-Imine´s second reaction run afforded an additional 0.21 mmol of alcohol product attributed to the second cycle, as the relation of signals was doubled after the second catalytic cycle.

Table S3: Crude-reaction analyses from the leaching experiments

Catalyst	Catalytic runs	Integration relation between signals (TMB (3H) : Product (1H)	mmol of product in the crude	Δmmol after filtering
COM-Imine	1	0.87 : 1	0.27	0.21
	2	0.48 : 1	0.48	
COM-Amine	1	0.97 : 1	0.24	0.01
	2	0.92 : 1	0.25	

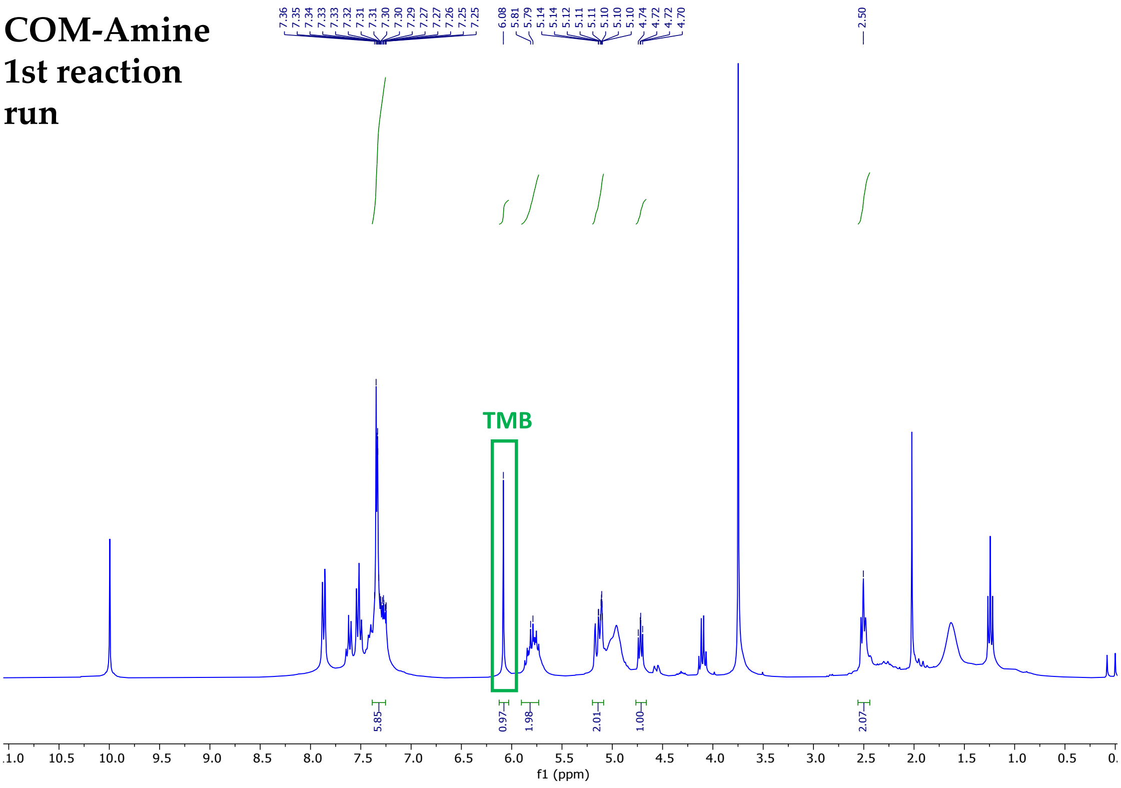

Figure S2: ^{1}HNMR spectrum of the crude mixture from the first catalytic cycle of the leaching experiments with the COM-Amine material.

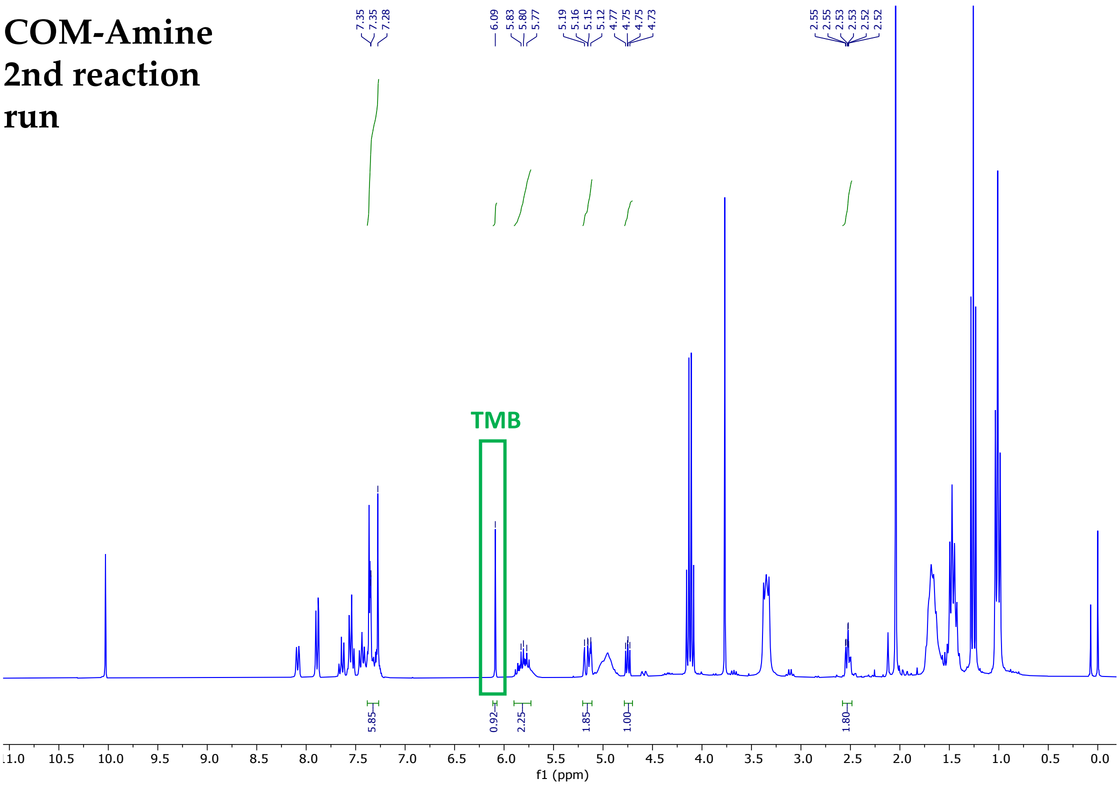

Figure S3: ^{1}HNMR spectrum of the crude mixture from the second catalytic cycle of the leaching experiments with the COM-Amine material.

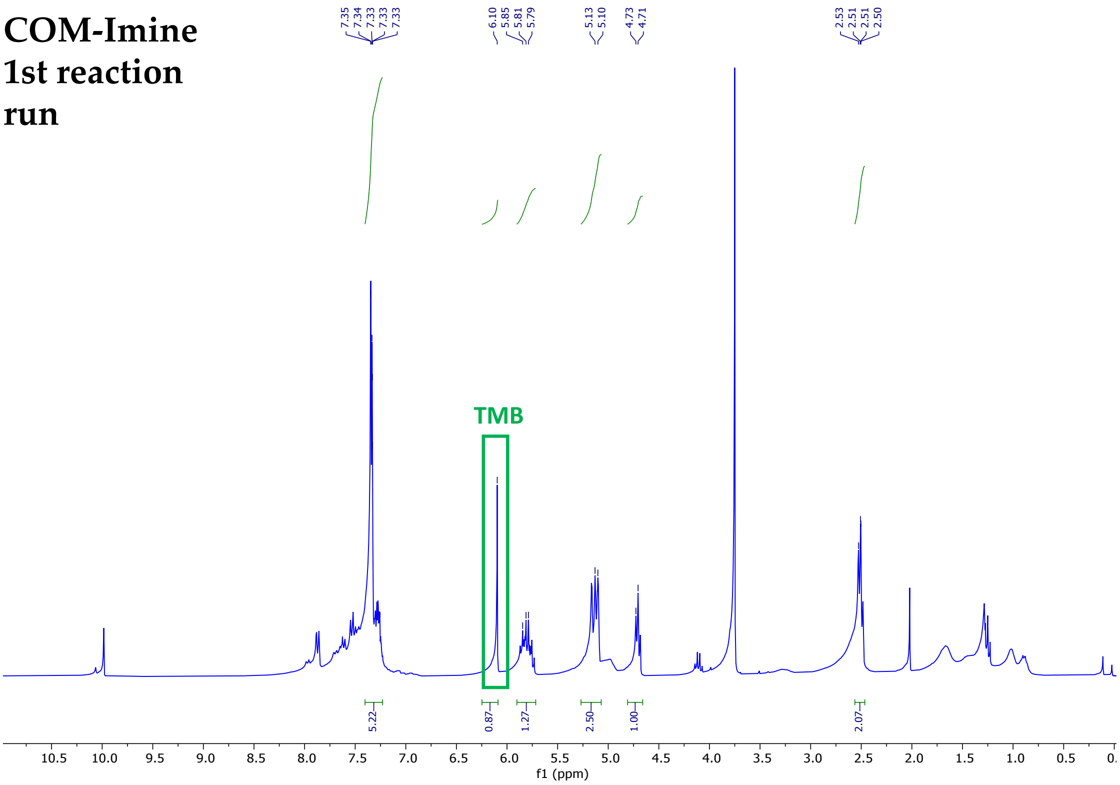

Figure S4: ^{1}HNMR spectrum of the crude mixture from the first catalytic cycle of the leaching experiments with the COM-Imine material.

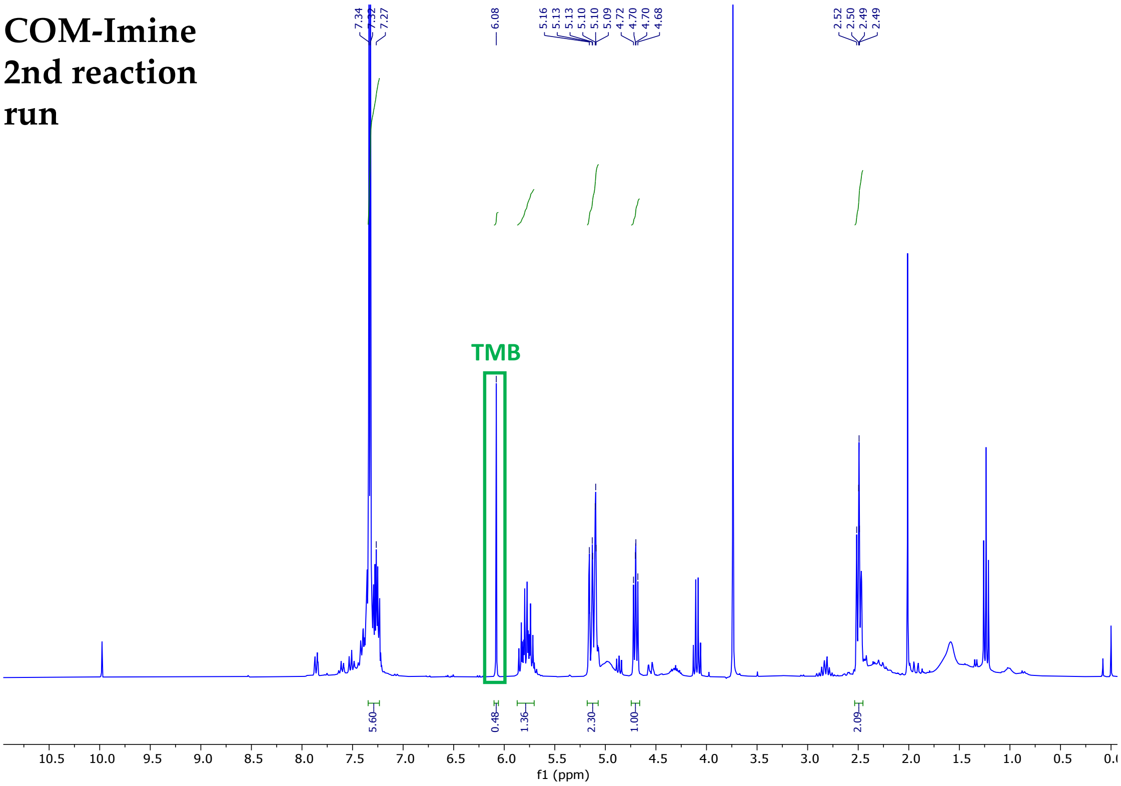

Figure S5: ^{1}HNMR spectrum of the crude mixture from the second catalytic cycle of the leaching experiments with the COM-Imine material.

7. Recyclability experiments

The recyclability of the COM-Amine material was studied by separating the solid catalyst from the crude reaction mixture of the model reaction with benzaldehyde by centrifugation (6000 rpm, rt) after each catalytic run. The reaction was not quenched after each cycle with 1 mL of 10% w/w NaOH solution. Then, the COM-Amine catalyst was washed 2 times with 5 mL of AcOEt, collecting and combining the organic phases with the main crude mixture for their work-up, and finally the catalyst was dried under vacuum overnight before the next catalytic cycle. The crude mixtures from each reaction were analyzed by ^{1}H-NMR and SFC in order to obtain values for the yield and enantiomeric ratio after each cycle. The retention times of the enantiomers in the chromatograms may vary, depending on the settings employed for each crude, in order to separate the signals of each enantiomer as much as possible.

Table S4: Yields and enantiomeric excesses from the recyclability experiments with COM-Amine catalyst

Catalytic run	Yield (%)	ee (%)
1	54	41
2	50	44
3	53	40
4	44	42
5	**23**	42

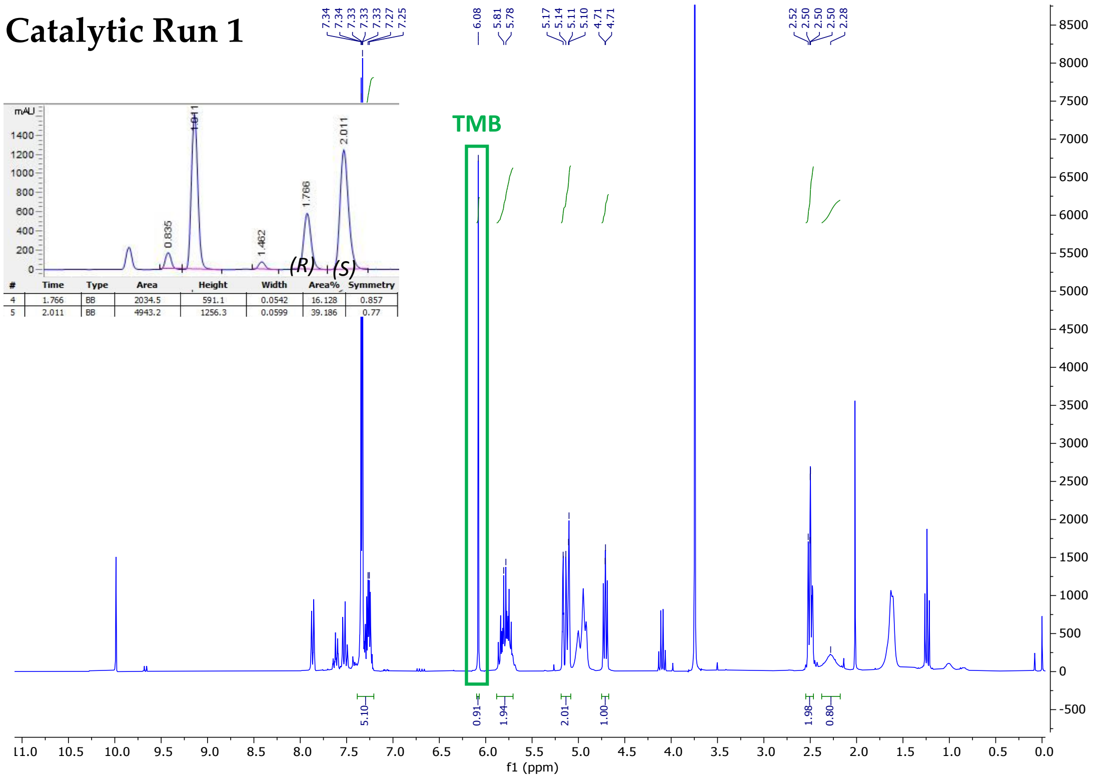

*Figure S6: ^{1}H-NMR and SFC-Chromatogram of the crude reaction mixture from the **first** catalytic run of the recyclability experiments. SFC Conditions: IB-Column, isocratic 5 % MeOH in supercritical CO_2, 10 min.*

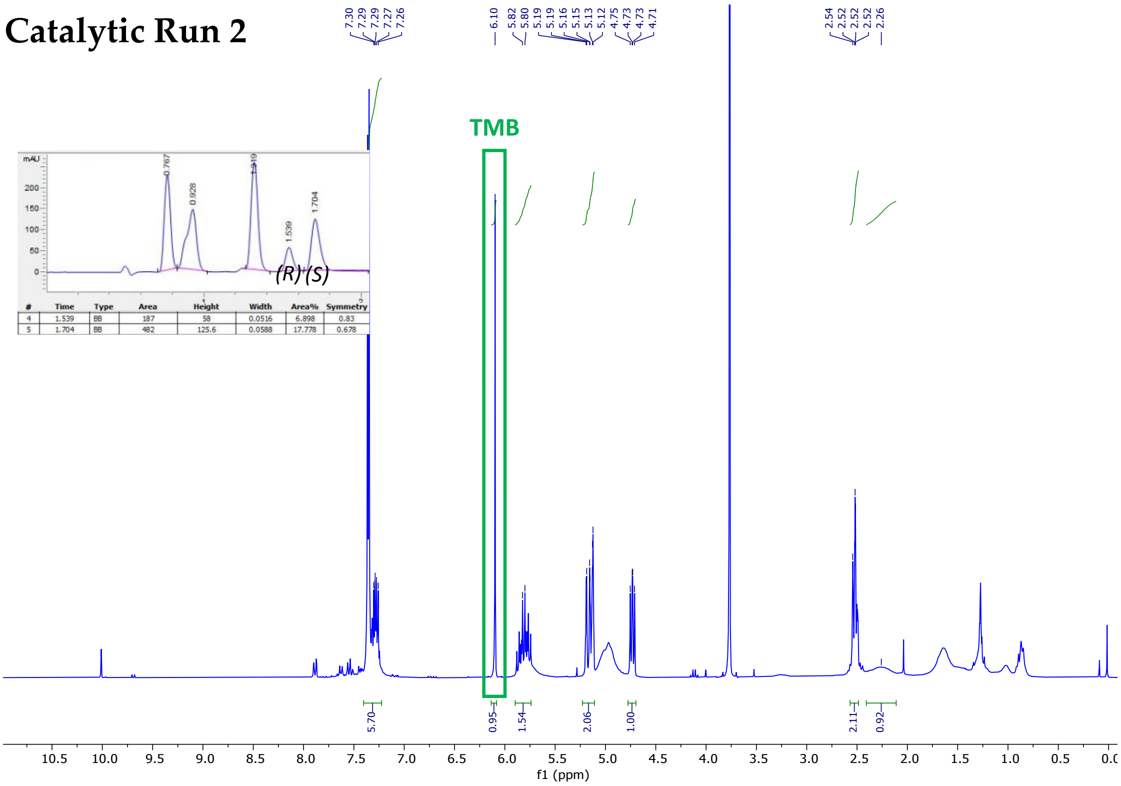

*Figure S7: ^{1}H-NMR and SFC-Chromatogram of the crude reaction mixture from the **second** catalytic run of the recyclability experiments. SFC Conditions: IB-Column, gradient 5-30% MeOH in supercritical CO_2, 10 min.*

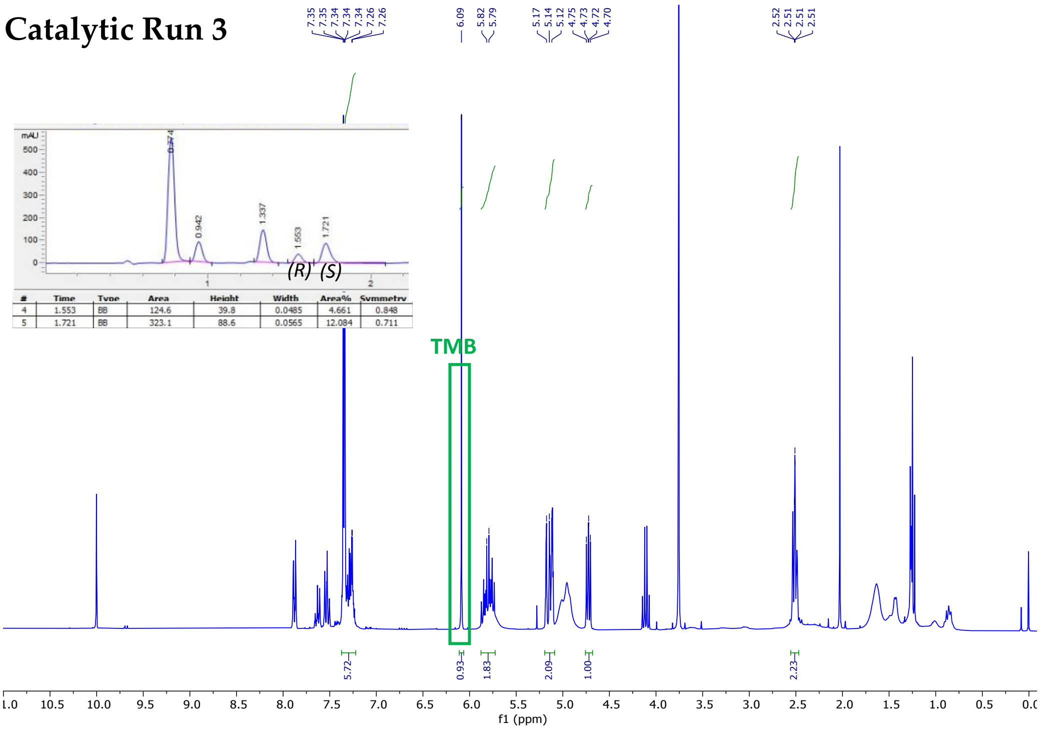

*Figure S8: ^{1}H-NMR and SFC-Chromatogram of the crude reaction mixture from the **third** catalytic run of the recyclability experiments. SFC Conditions: IB-Column, gradient 5-30% MeOH in supercritical CO_2, 10 min.*

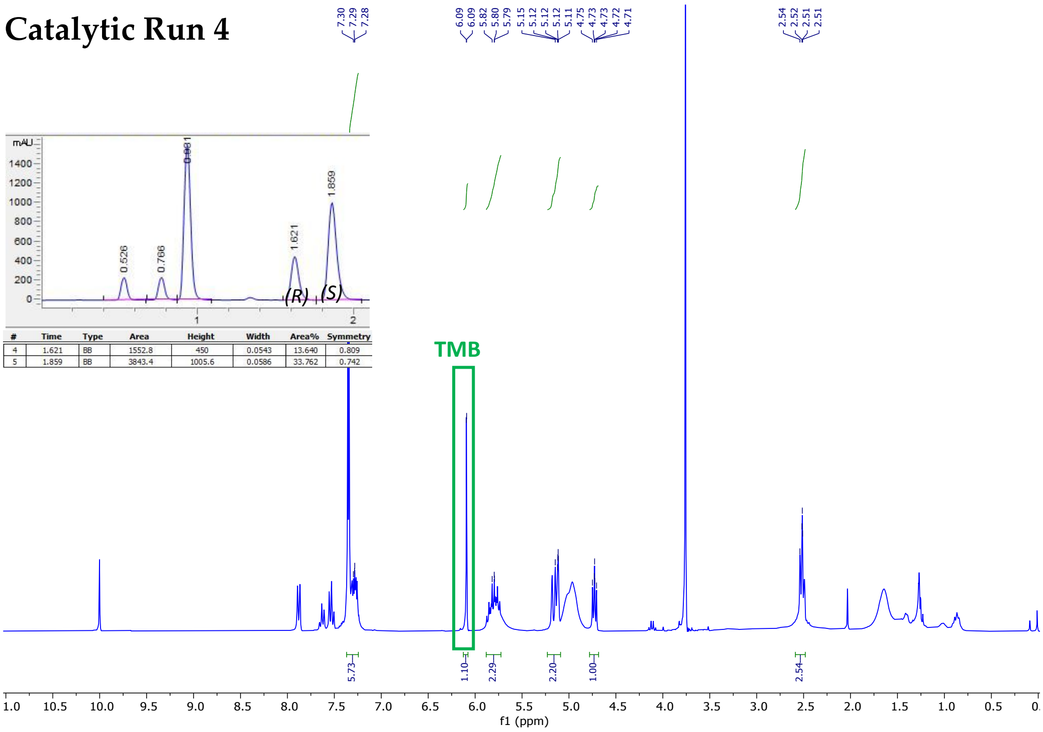

*Figure S9: ^{1}H-NMR and SFC-Chromatogram of the crude reaction mixture from the **fourth** catalytic run of the recyclability experiments. SFC Conditions: IB-Column, isocratic 5% MeOH in supercritical CO_2, 10 min.*

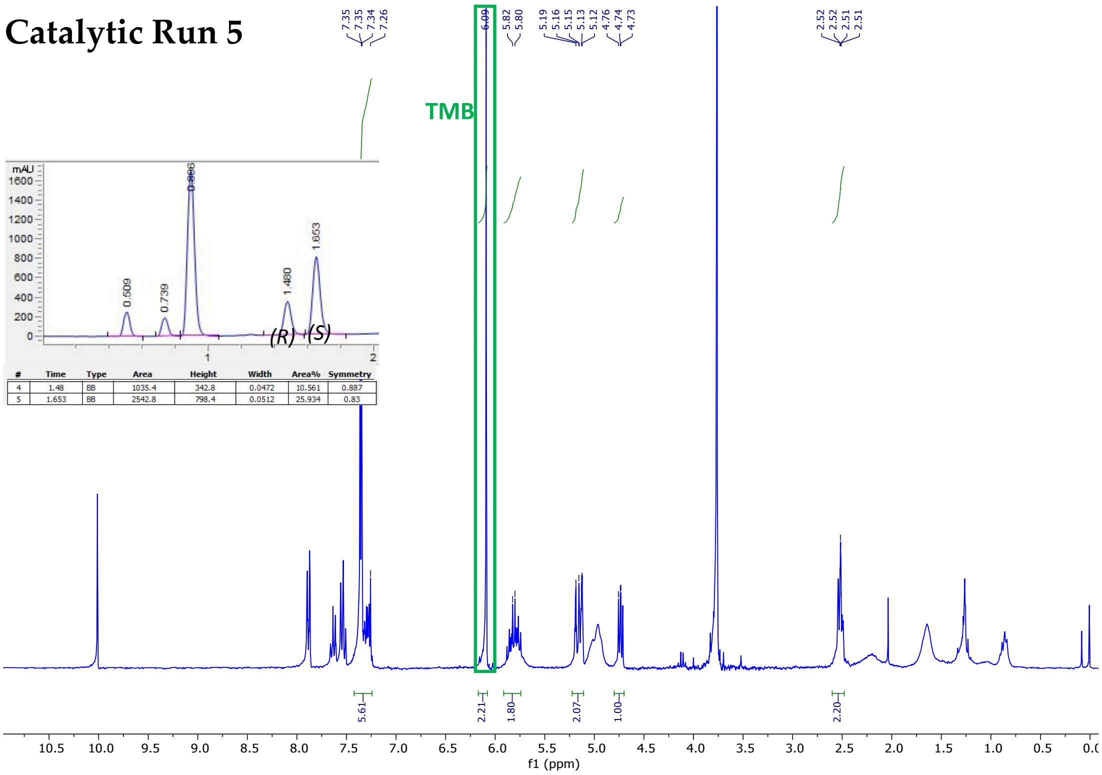

*Figure S10: ^{1}H-NMR and SFC-Chromatogram of the crude reaction mixture from the **fifth** catalytic run of the recyclability experiments. SFC Conditions: IB-Column, gradient 5-30% MeOH in supercritical CO_2, 10 min.*

8. ^{1}H-NMR and ^{31}P-NMR spectra of building unit´s precursors (1 and 2) and building unit (3) ($CDCl_3$, 300 MHz for ^{1}H and 500 MHz for ^{31}P)

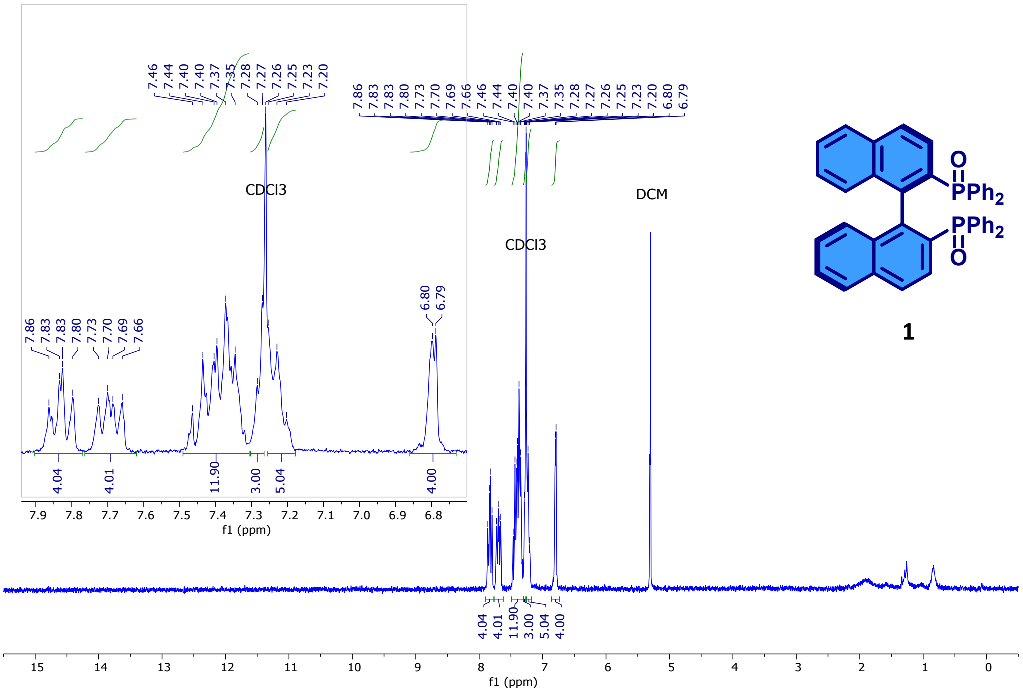

*Figure S11: ^{1}H-NMR spectrum of **1***

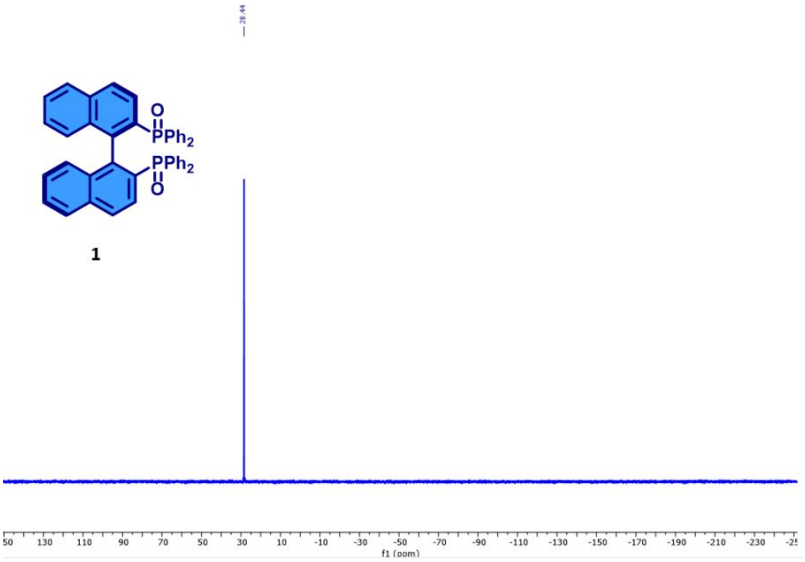

*Figure S12: ^{31}P-NMR spectrum of **1***

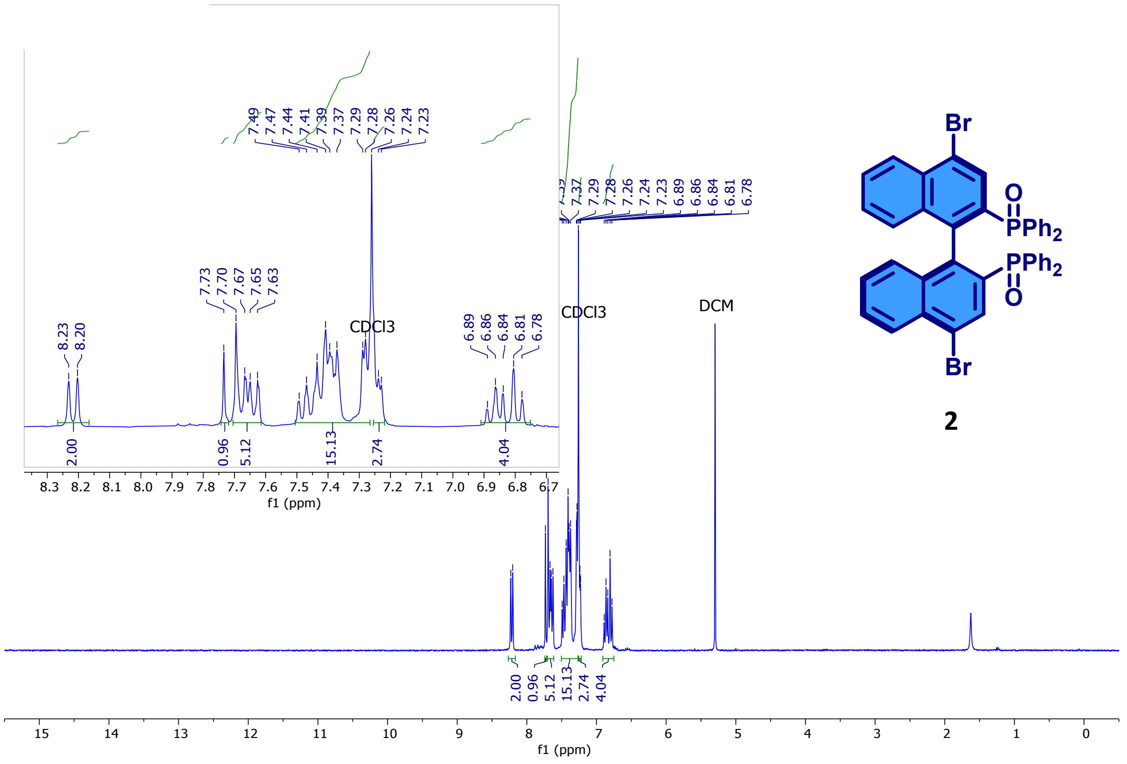

*Figure S13: ^{1}H-NMR spectrum of **2***

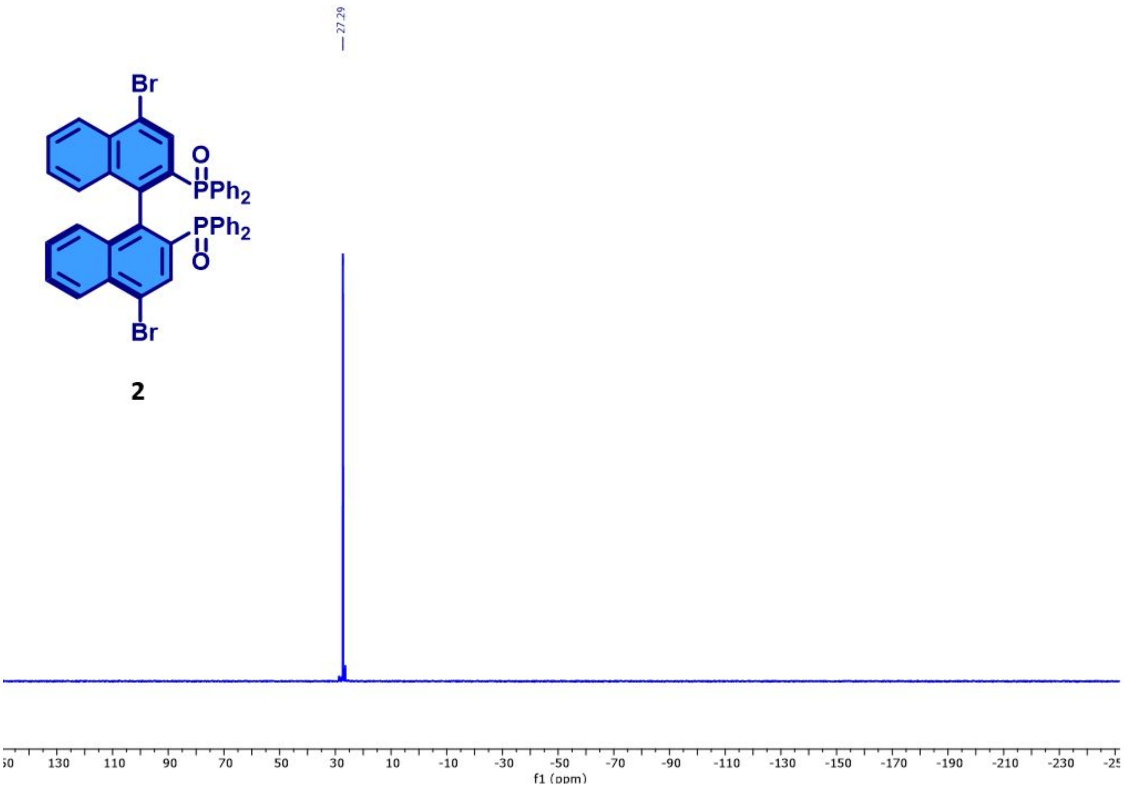

*Figure S14: ^{31}P-NMR spectrum of **2***

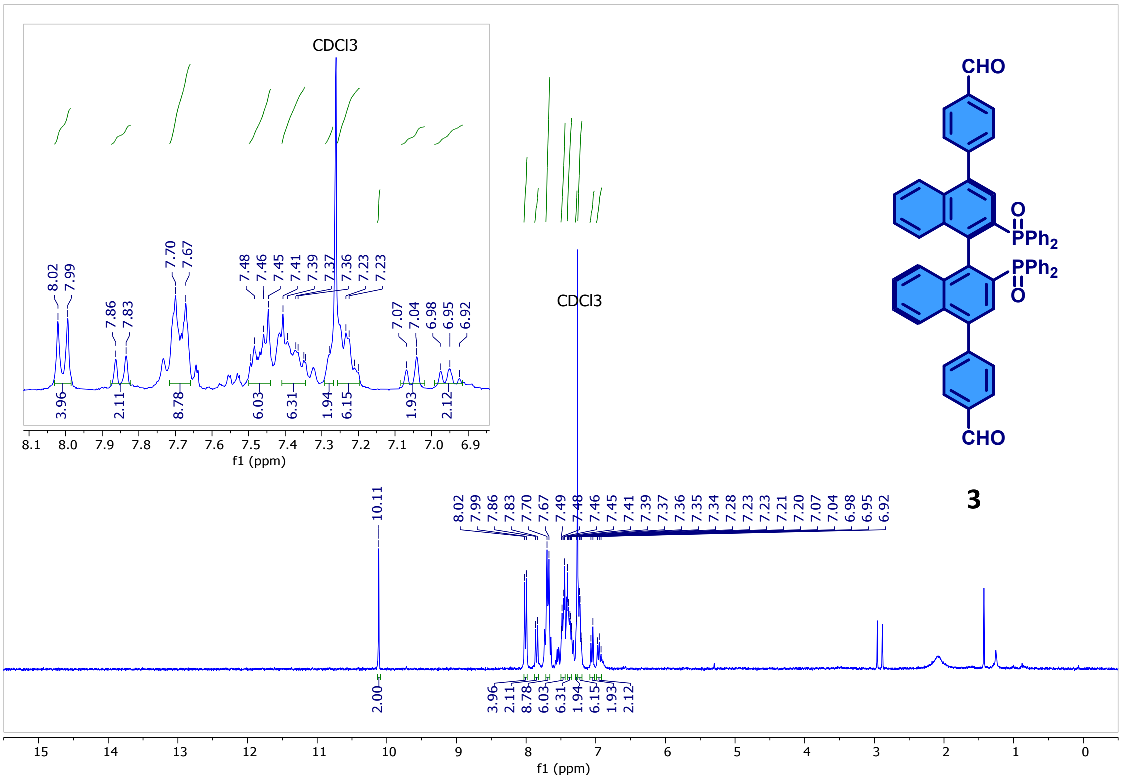

Figure S15: ^{1}H-NMR spectrum of **3**

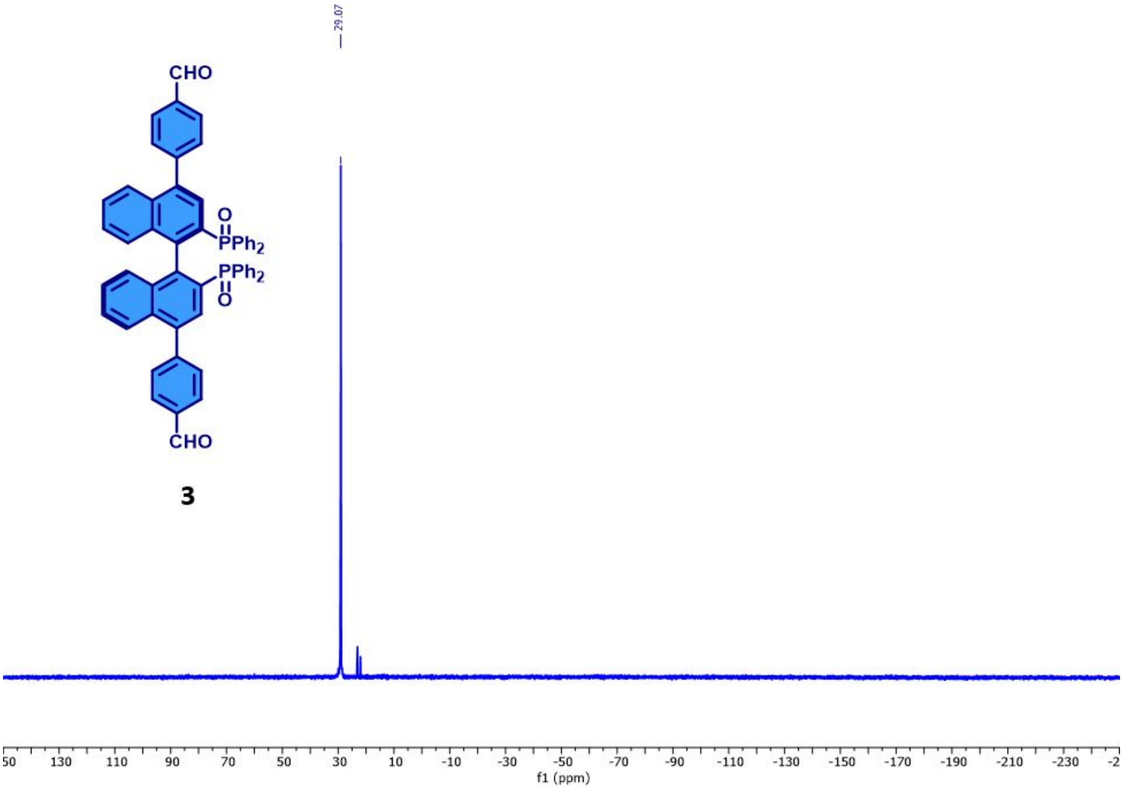

Figure S16: ^{31}P-NMR spectrum of **3**

9. MALDI-TOF-mass spectra of building unit´s precursors (1 and 2) and building unit (3)

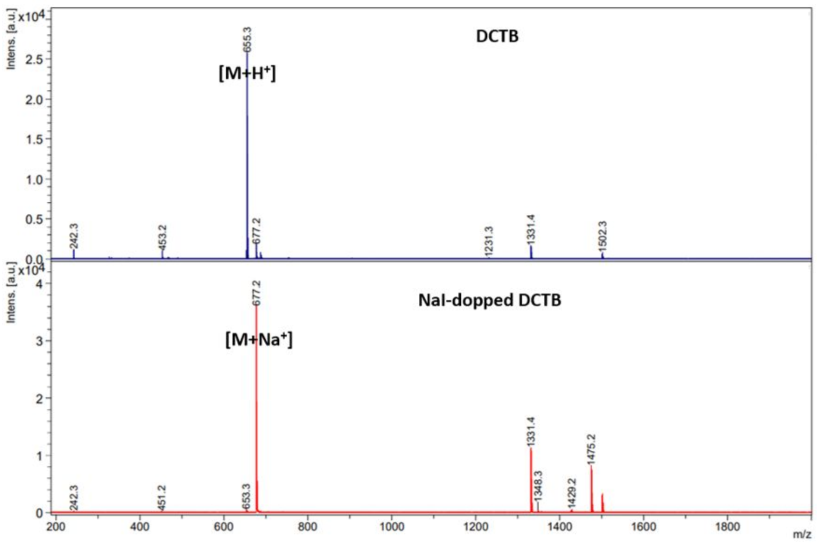

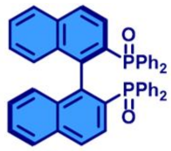

Molecular Weight: 654,69

1

*Figure S17: MALDI-TOF-mass spectrum of **1***

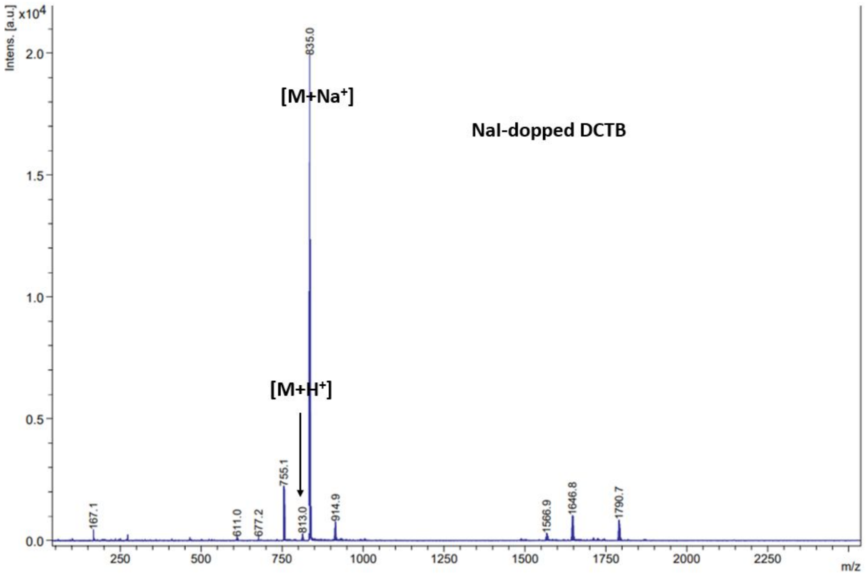

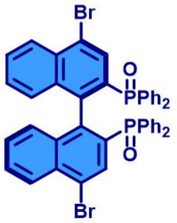

Molecular Weight: 812,48

2

*Figure S18: MALDI-TOF-mass spectrum of **2***

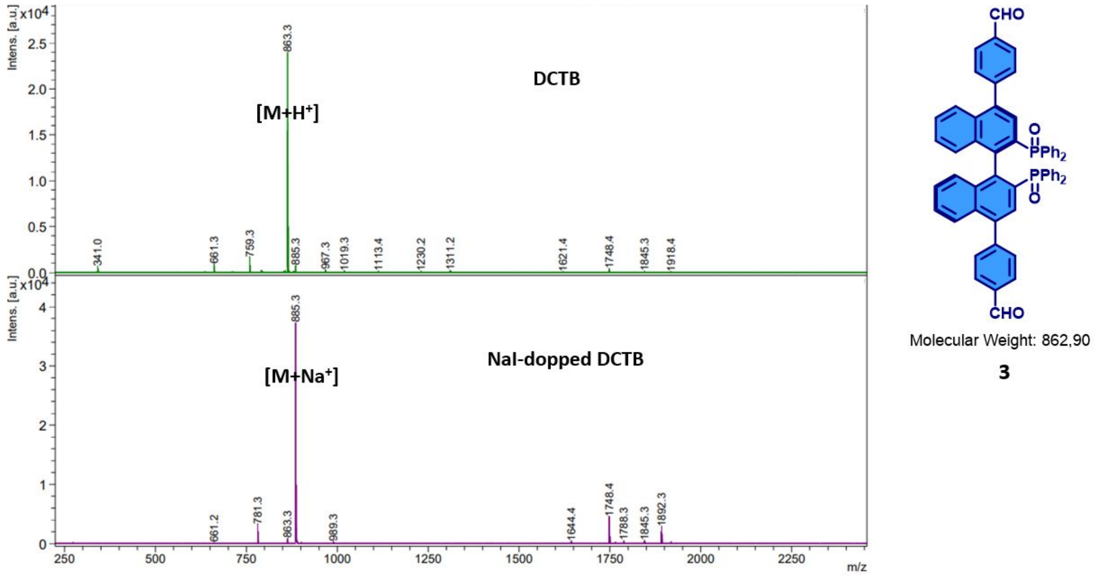

*Figure S19: MALDI-TOF-mass spectrum of **3***

10. FT-IR spectra of the building unit **3** and TAPB

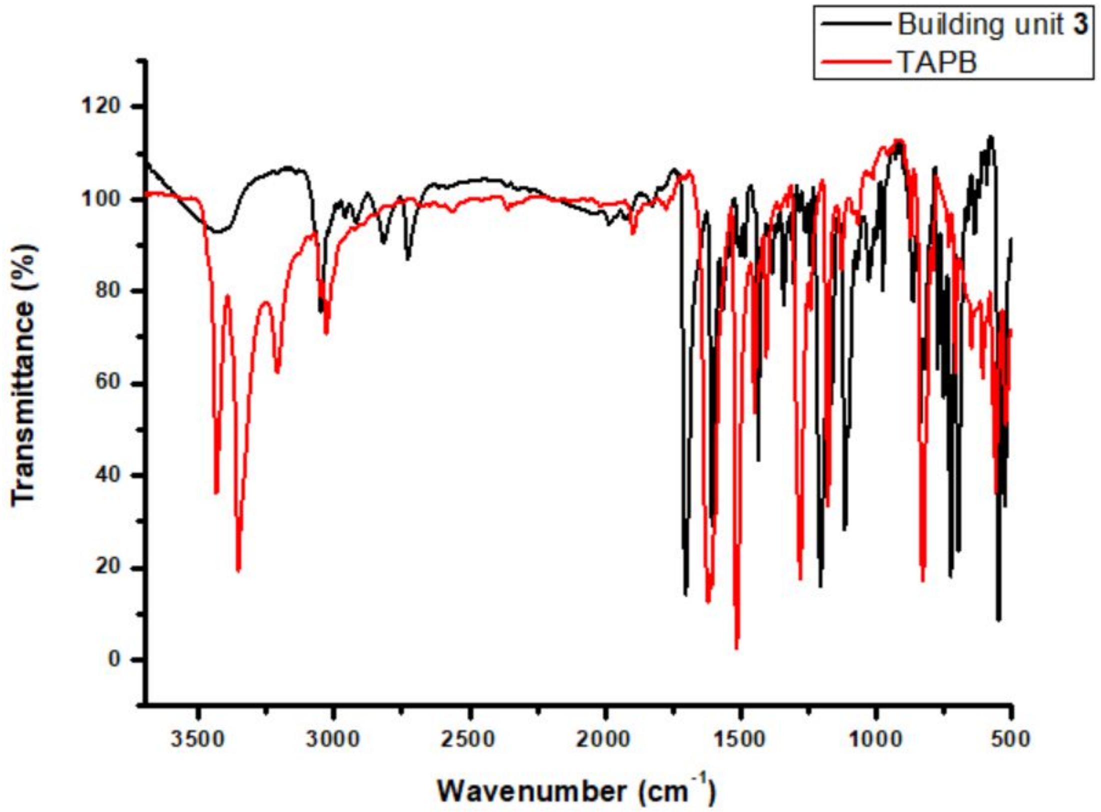

Figure S20: FT-IR spectra of the building unit ***3*** *(black) and 1,3,5-tris(aminophenyl)benzene* ***TAPB*** *(red)*

11. Thermogravimetric analyses of COM-Imine and COM-Amine materials

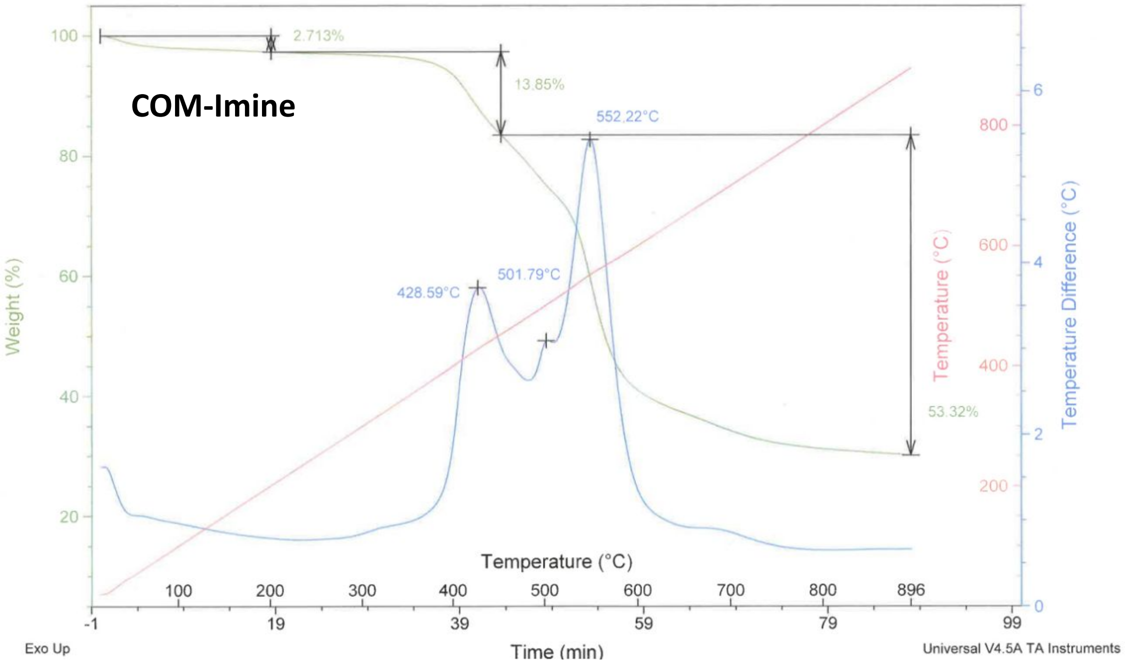

Figure S21: Thermogravimetric analysis of COM-Imine

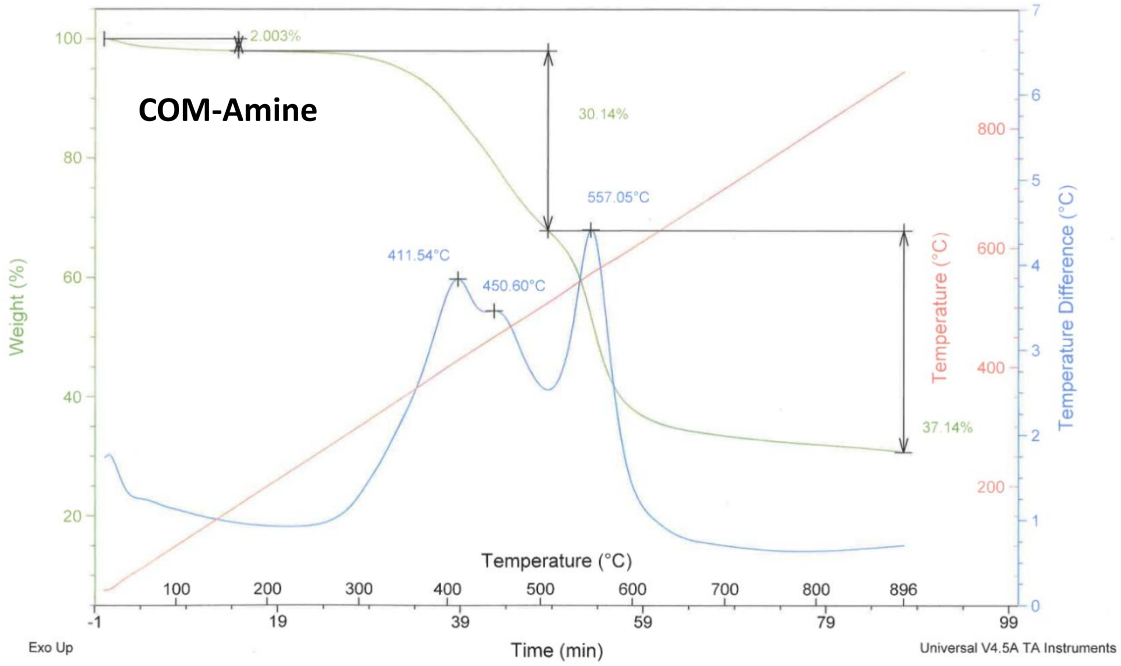

Figure S22: Thermogravimetric analysis of COM-Amine

12. N_2 adsorption-desorption isotherms of COM-Imine and COM-Amine materials

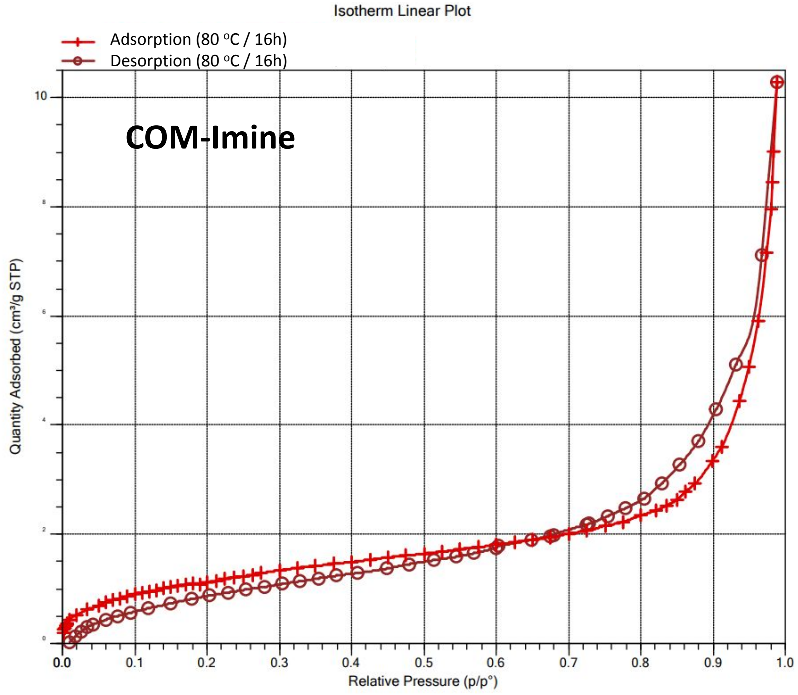

Figure S23: N_2 adsorption-desorption Isotherm of COM-Imine

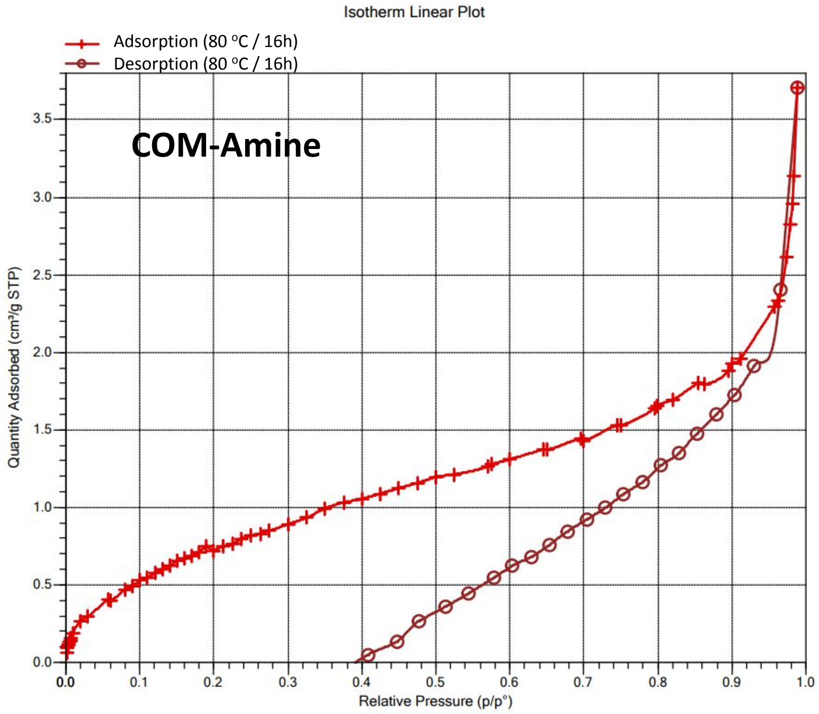

Figure S24: N_2 adsorption-desorption Isotherm of COM-Amine

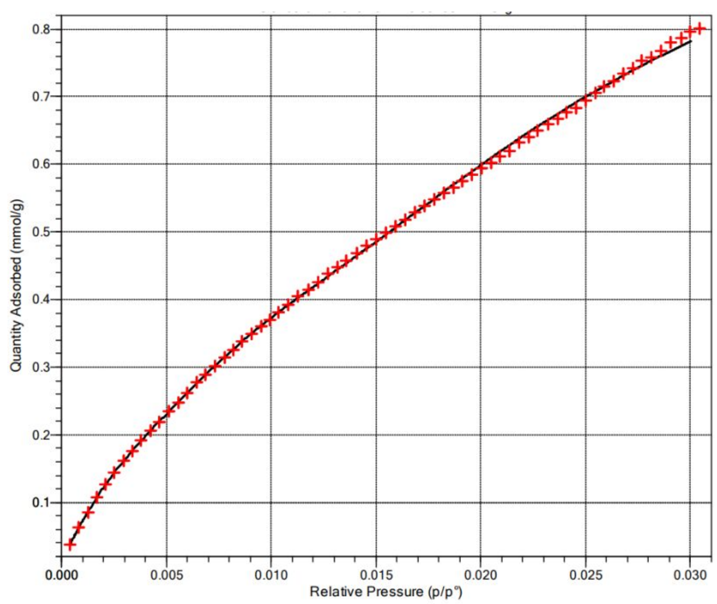

Figure S25: CO_2 adsorption isotherm of COM-Amine

13. ^{31}P-NMR spectra of COM-Imine and COM-Amine materials

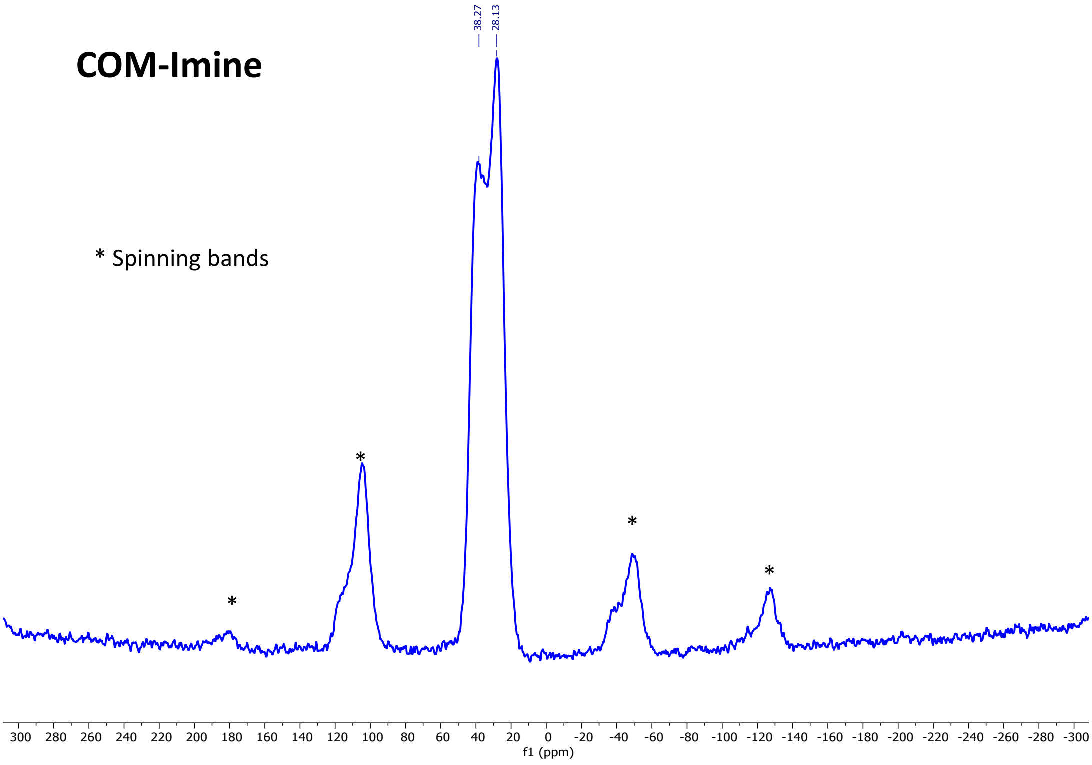

Figure S26: Solid-state ^{31}P-NMR spectrum of COM-Imine

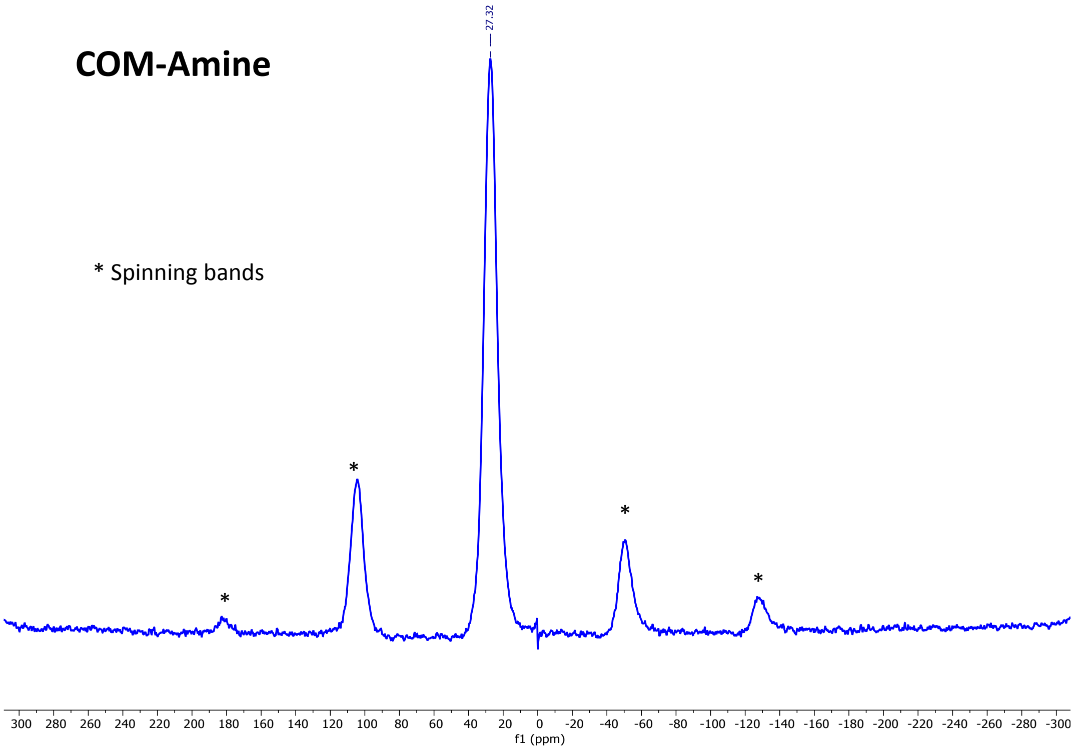

Figure S27: Solid-state ^{31}P-NMR spectrum of COM-Amine

14. X-ray diffraction patterns from COM-Imine and COM-Amine materials

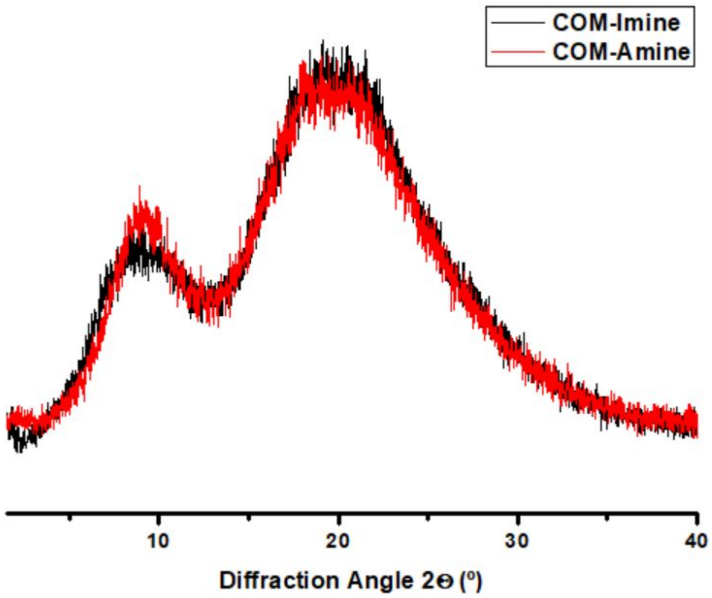

Figure S28: Comparative powder-XRD analysis of COM-Imine and COM-Amine

15. X-ray photoelectron spectra of COM-Imine and COM-Amine

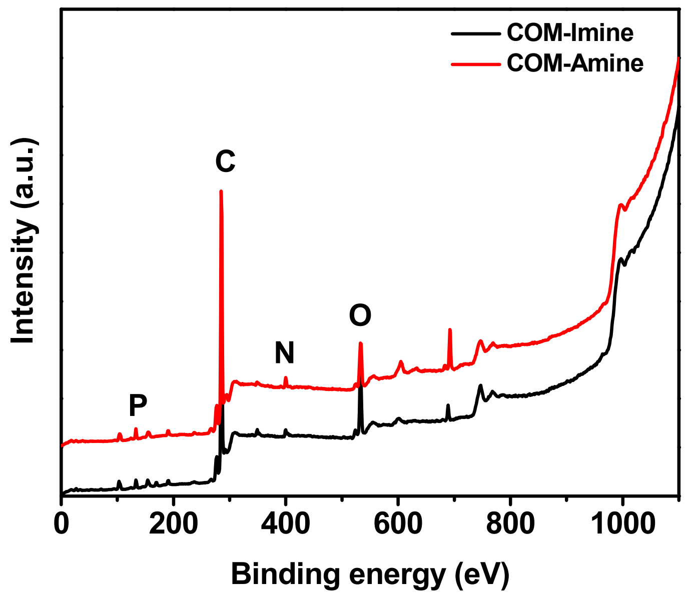

Figure S29: XPS spectra of COM-Imine and COM-Amine

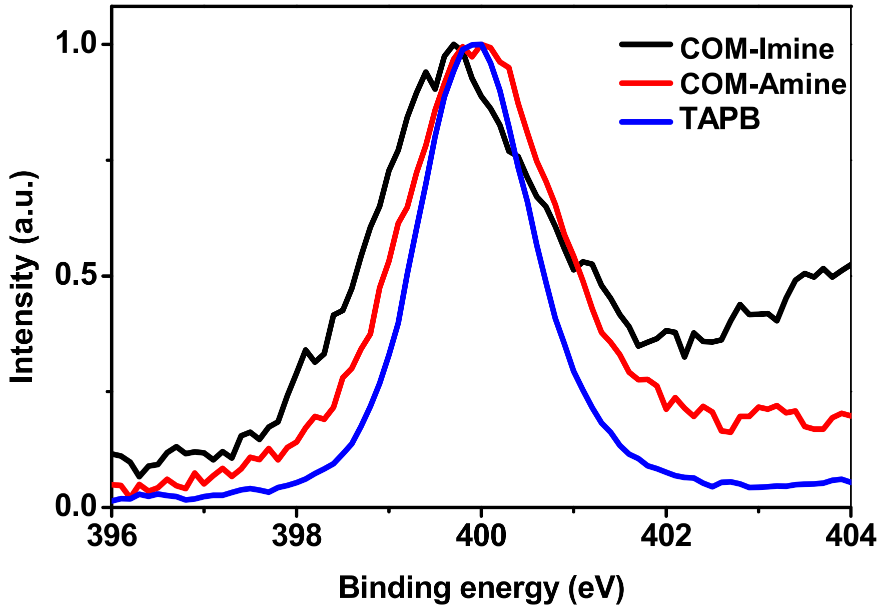

Figure S30: N 1s region of XPS spectrum of COM-Imine, COM-Amine and TAPB

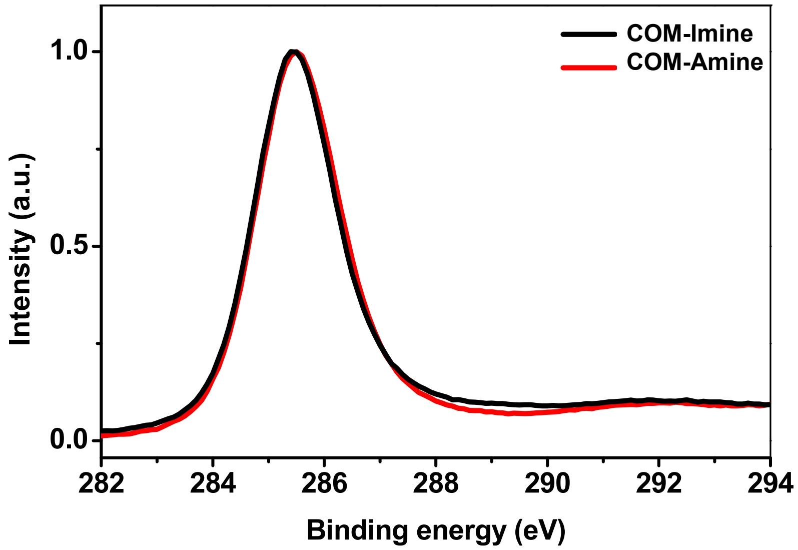

Figure S31: C 1s region of XPS spectrum of COM-Imine and COM-Amine

16. FT-IR spectrum of COM-Imine and COM-Amine materials

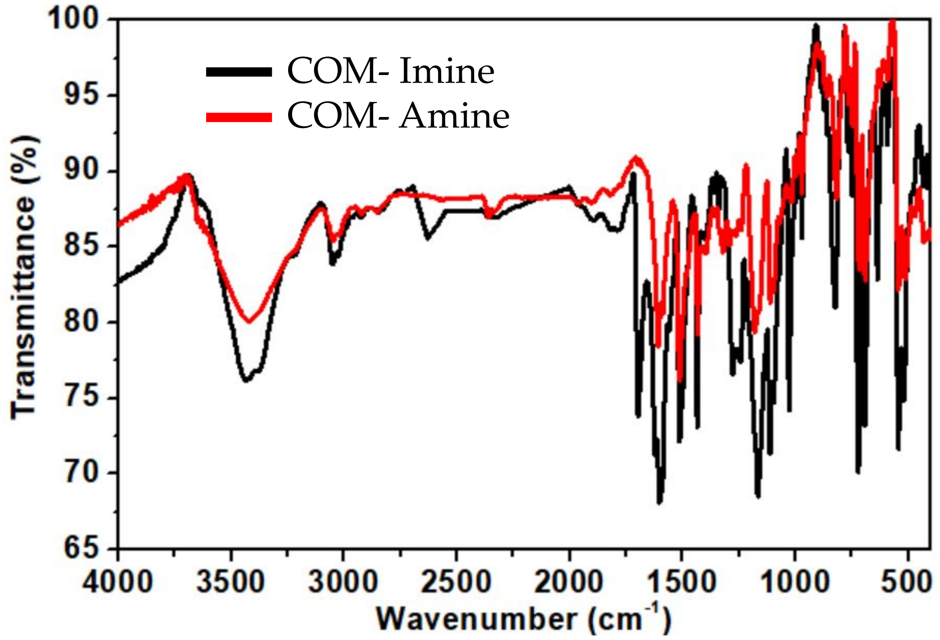

Figure S32: FT-IR spectra of COM-Imine and COM-Amine materials

17. Characterization of the products of the aldol addition reactions.

The integrated signals shown in the spectra correspond to each product (except from those signals indicated in green, corresponding to the 1,3,5-tri(methoxy)benzene, the internal standard).

1-phenylbut-3-en-1-ol (4a)[3]:

^{1}H-NMR: δ 7.34-7.25 (m, 5H), 5.81-5.78 (m, 1H), 5.17-5.10 (m, 2H), 4.71 (m, 1H), 2.51 (m, 2H), 2.28 (s, 1H, -OH).

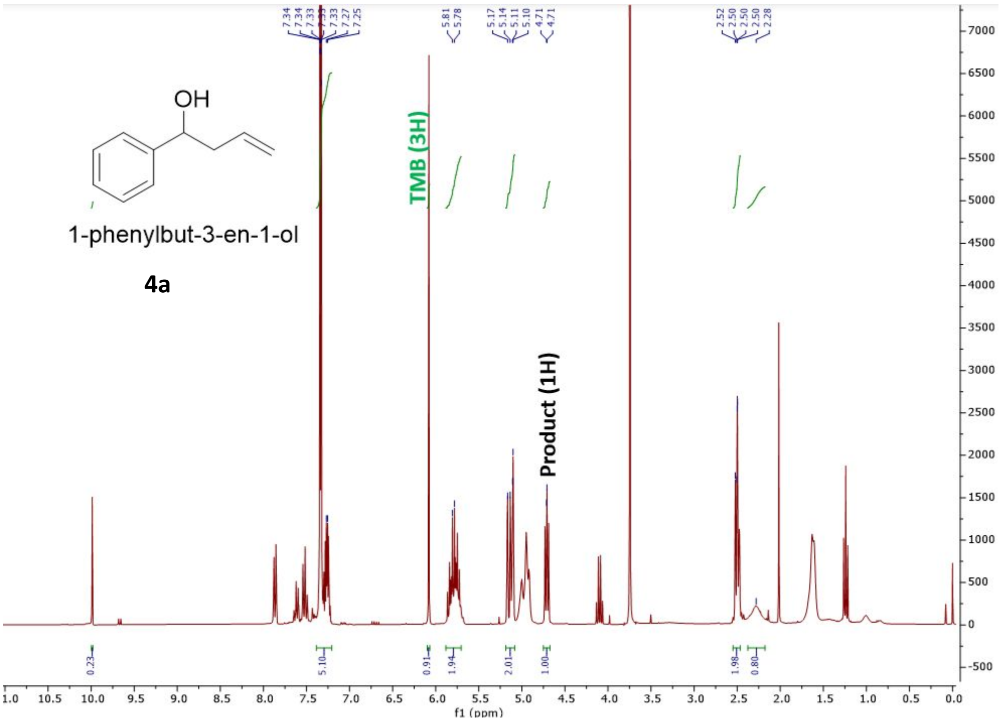

*Figure S33: ^{1}H-NMR spectrum of the crude reaction mixture containing product **4a.***

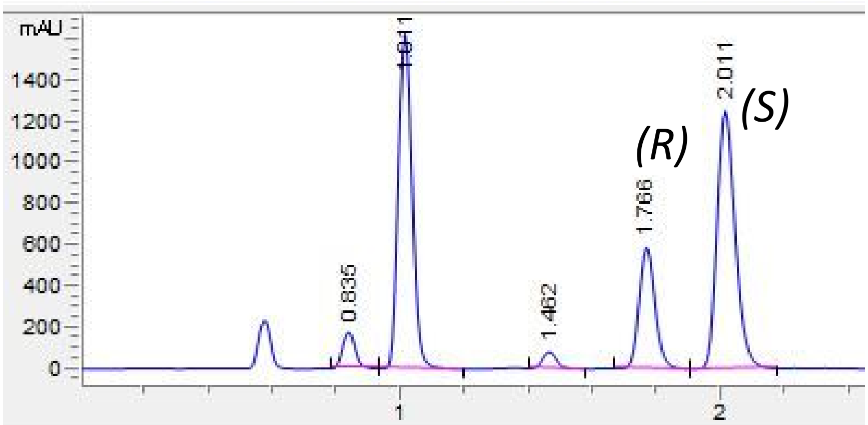

Time	Type	Area	Height	Width	Area%	Symmetry
1.766	BB	2034.5	591.1	0.0542	16.128	0.857
2.011	BB	4943.2	1256.3	0.0599	39.186	0.77

*Figure S34: SFC-Chromatogram of the crude reaction mixture containing product **4a.** SFC Conditions: IB-Column, isocratic 5 % MeOH in supercritical CO_2, 10 min.*

1-(4-chlorophenyl)but-3-en-1-ol (4b)[4]:

^{1}H-NMR**:** δ 7.30-7.24 (m, 4H), 5.80-5.71 (m, 1H), 5.14-5.09 (m, 2H), 4.68 (m, 1H), 2.75 (s, 1H, -OH), 2.45 (m, 2H).

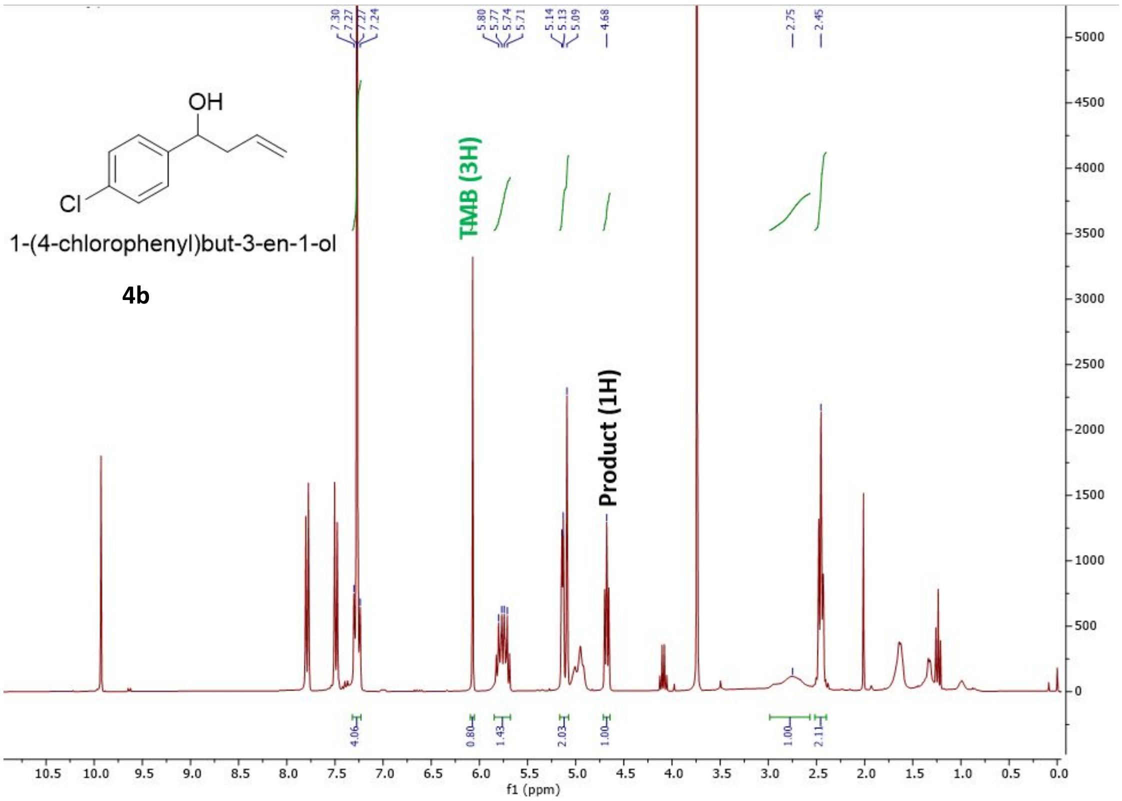

*Figure S35: ^{1}H-NMR spectrum of the crude reaction mixture containing product **4b.***

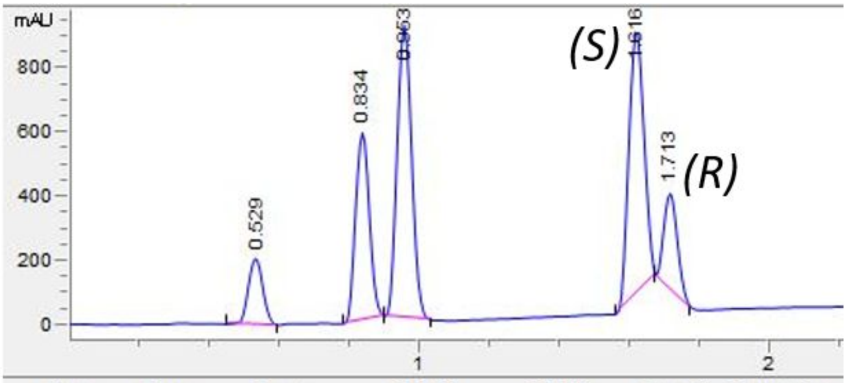

Time	Type	Area	Height	Width	Area%	Symmetry
1.616	BB	2492.6	828.4	0.0491	30.762	0.939
1.713	BB	782.6	296	0.0428	9.659	0.806

*Figure S36: SFC-Chromatogram of the crude reaction mixture containing product **4b.** SFC Conditions: ID-Column, gradient 5-40 % MeOH in supercritical CO_2, 10 min.*

1-(3,5-dimethylphenyl)but-3-en-1-ol (4c)[5]:

^{1}H-NMR**:** δ 6.99 (s, 2H), 6.93 (s, 1H), 5.84-5.82 (m, 1H), 5.20-5.13 (m, 2H), 4.66 (m, 1H), 2.51 (m, 2H), 2.33 (s, 6H).

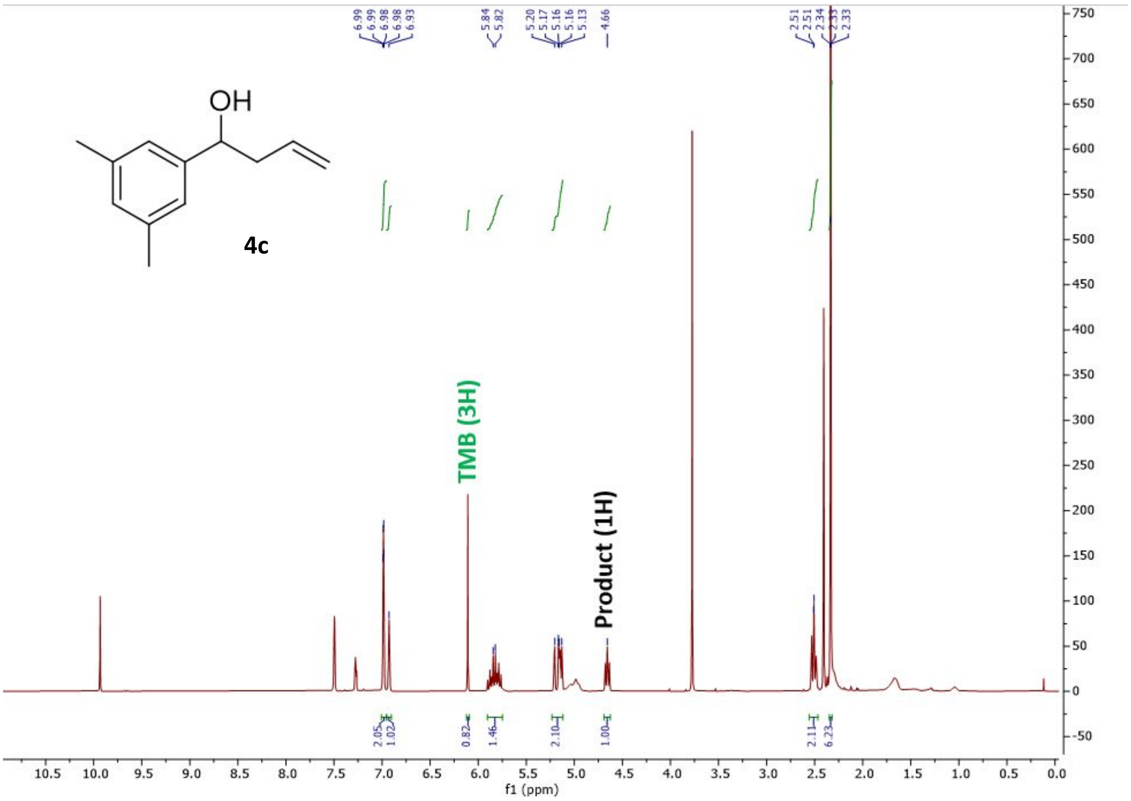

*Figure S37: ^{1}H-NMR spectrum of the crude reaction mixture containing product **4c.***

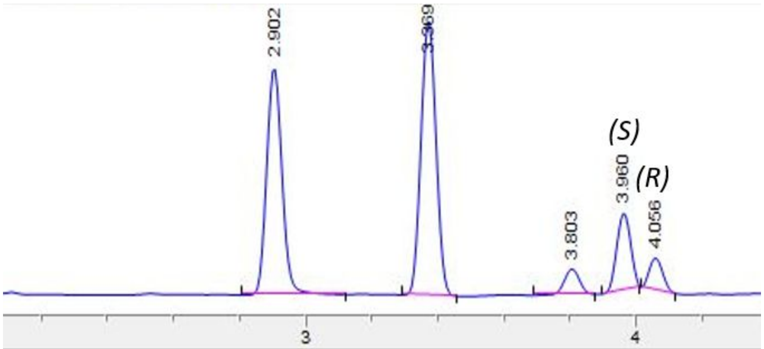

Time	Type	Area	Height	Width	Area%	Symmetry
3.96	BB	743.5	254.2	0.0481	11.302	1.019
4.056	BB	293	104.2	0.0448	4.454	0.825

*Figure S38: SFC-Chromatogram of the crude reaction mixture containing product **4c.** SFC Conditions: IG-Column, gradient 5-40 % MeOH in supercritical CO_2, 10 min.*

1-(3,5-bis(trifluoromethyl)phenyl)but-3-en-1-ol (4d):

^{1}H-NMR**:** δ 7.81 (s, 2H), 7.73 (s, 1H), 5.80-5.71 (m, 1H), 5.16-5.12 (m, 2H), 4.92 (m, 1H), 3.21 (s, 1H, -OH), 2.52-2.48 (m, 2H).

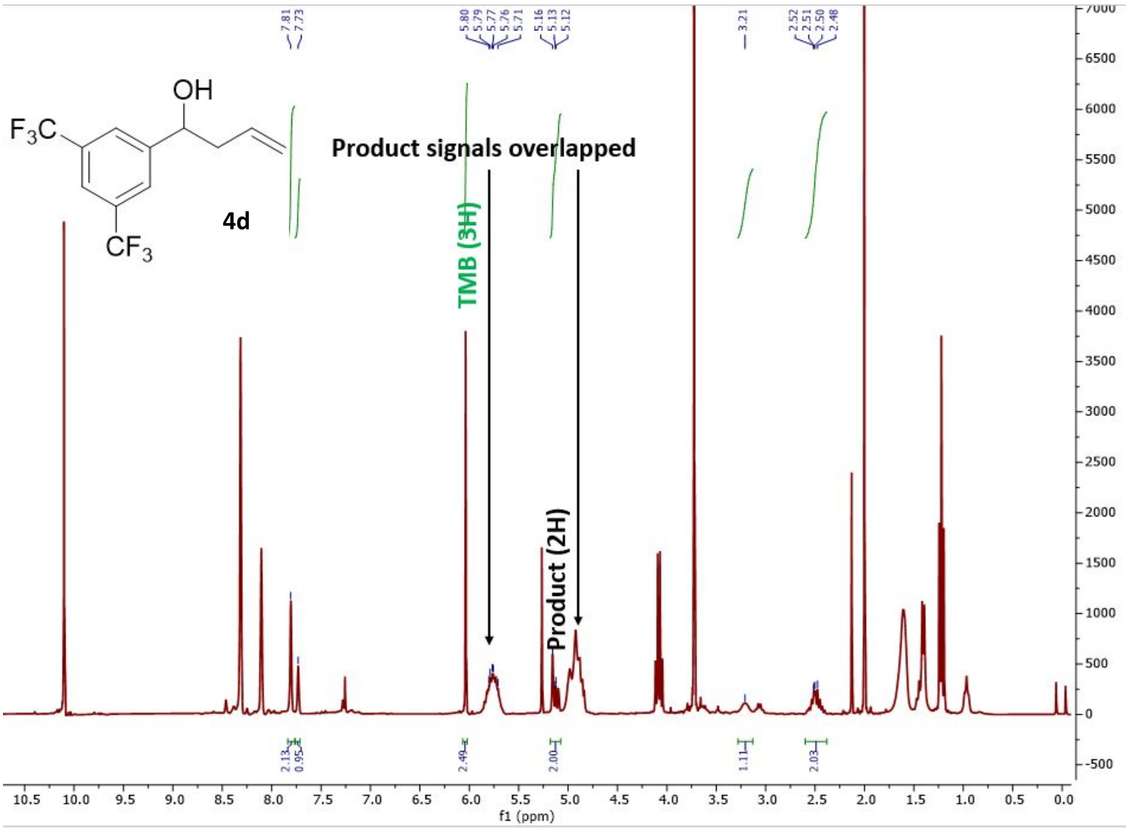

*Figure S39: ^{1}H-NMR spectrum of the crude reaction mixture containing product **4d.***

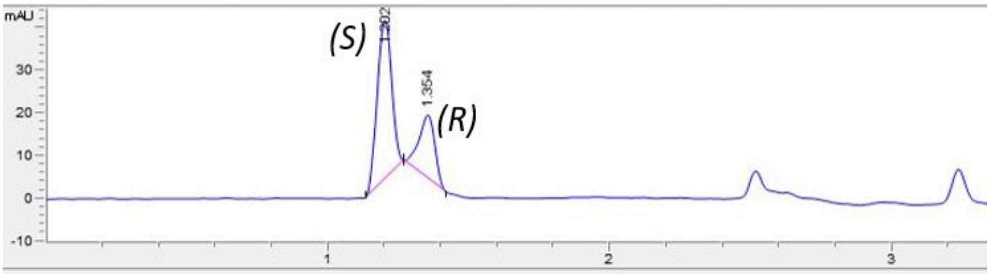

Time	Type	Area	Height	Width	Area%	Symmetry
1.202	BB	135	37.4	0.0561	69.990	1.095
1.354	BB	57.9	15	0.0572	30.010	1.223

*Figure S40: SFC-Chromatogram of the crude reaction mixture containing product **4d.** SFC Conditions: IG-Column, gradient 5-40 % MeOH in supercritical CO_2, 10 min.*

1-(3,4,5-trimethoxyphenyl)but-3-en-1-ol (4e)[6]:

^{1}H-NMR**:** δ 6.58 (s, 2H), 5.89-5.75 (m, 1H), 5.18-5.10 (m, 2H), 4.65 (m, 1H), 3.85 (s, 6H), 3.82 (s, 3H), 2.60 (s, 1H, -OH), 2.48 (m, 2H).

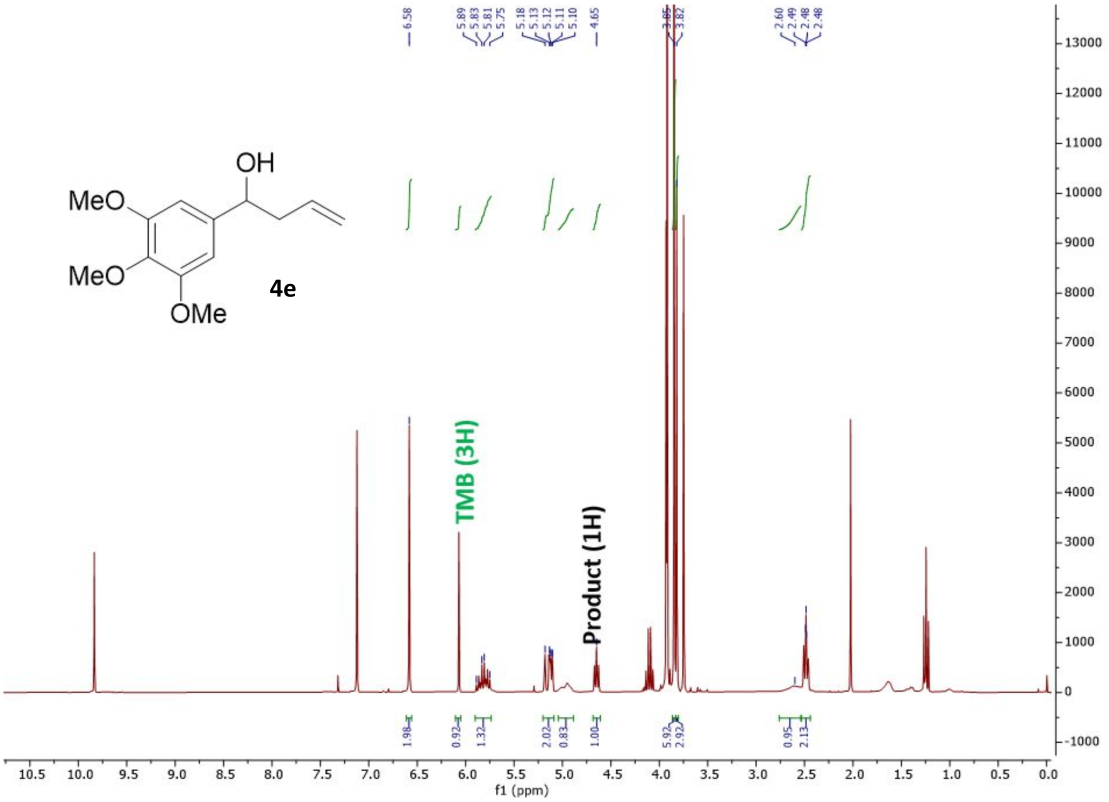

*Figure S41: ^{1}H-NMR spectrum of the crude reaction mixture containing product **4e.***

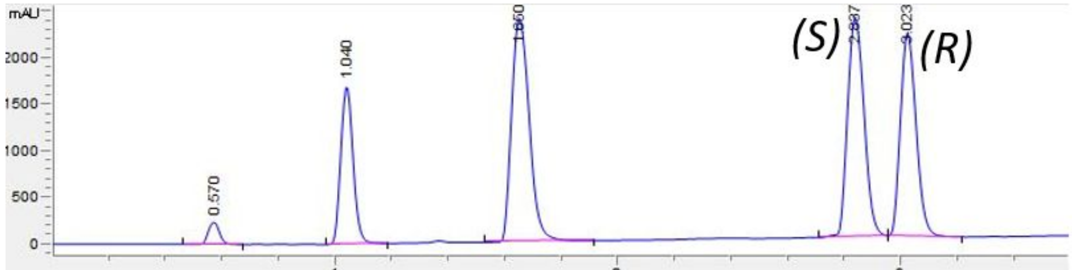

Time	Type	Area	Height	Width	Area%	Symmetry
2.837	BB	9509.5	2356.2	0.0651	27.921	0.794
3.023	BB	8351.9	2188.2	0.0625	24.522	0.773

*Figure S42: SFC-Chromatogram of the crude reaction mixture containing product **4e.** SFC Conditions: IB-Column, gradient 5-30 % MeOH in supercritical CO_2, 10 min.*

1-(4-iodophenyl)but-3-en-1-ol (4f):

^{1}H-NMR**:** δ 7.64 (d, *J* = 8.0 Hz, 2H), 7.08 (d, *J* = 8.0 Hz, 2H), 5.76-5.71 (m, 1H), 5.10 (m, 2H), 4.65 (m, 1H), 2.47-2.44 (m, 2H).

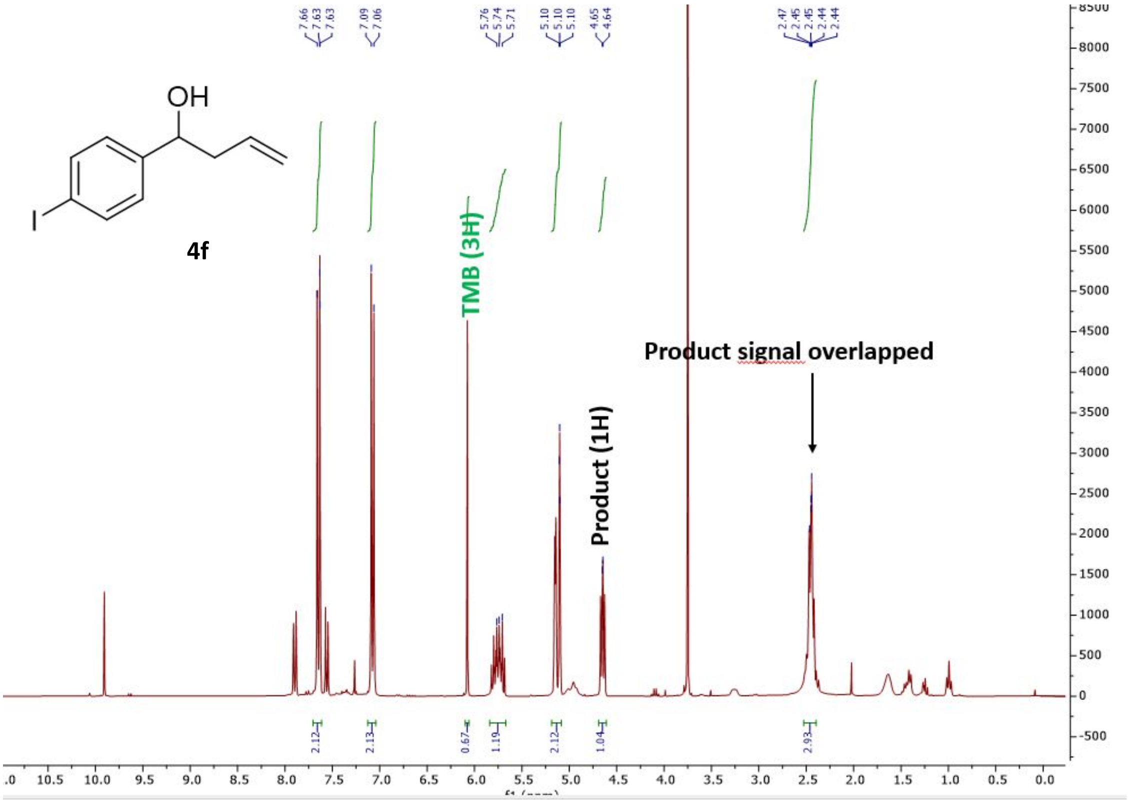

*Figure S43: ^{1}H-NMR spectrum of the crude reaction mixture containing product **4f.***

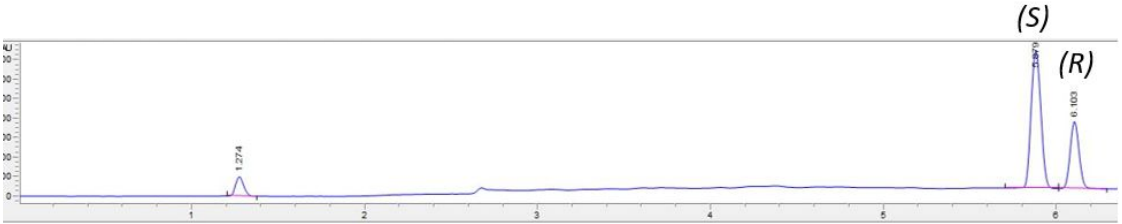

Time	Type	Area	Height	Width	Area%	Symmetry
5.879	BB	5547.9	1419.8	0.0636	64.032	0.872
6.103	BB	2467.3	692.8	0.0576	28.477	0.894

*Figure S44: SFC-Chromatogram of the crude reaction mixture containing product **4f.** SFC Conditions: IG-Column, gradient 5-40 % MeOH in supercritical CO_2, 10 min.*

1-(anthracen-9-yl)but-3-en-1-ol (4g)[7]:

^{1}H-NMR**:** δ 8.64-8.59 (m, 2H), 8.35 (s, 1H), 8.00-7.96 (m, 2H), 7.46 (m, 4H), 6.23 (m, 1H), 5.97-5.84 (m, 1H), 5.23(m, 1H), 5.11 (m, 1H), 2.81 (m, 2H).

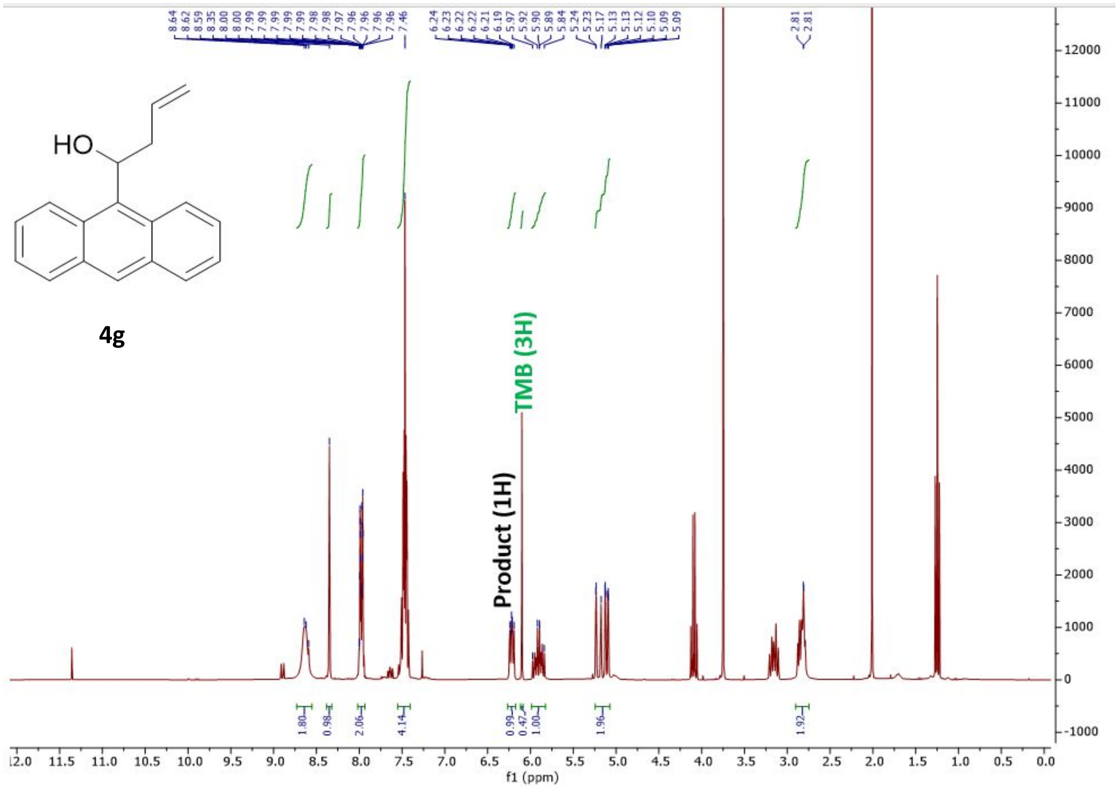

Figure S45: ^{1}H-NMR spectrum of the crude reaction mixture containing product ***4g.***

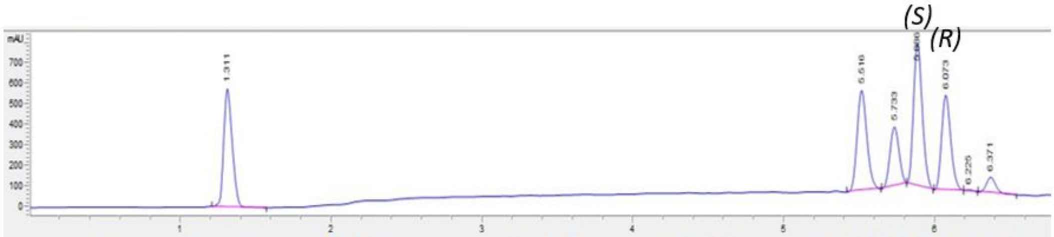

Time	Type	Area	Height	Width	Area%	Symmetry
5.886	BB	2847.2	703.7	0.0632	26.117	0.779
6.073	BB	1972.3	468.5	0.0651	18.092	0.797

Figure S46: SFC-Chromatogram of the crude reaction mixture containing product ***4g.*** *SFC Conditions: IA-Column, gradient 5-40 % MeOH in supercritical CO_2, 10 min.*

1-(naphthalen-1-yl)but-3-en-1-ol (4h)[8]:

^{1}H-NMR**:** δ 7.96 (m, 1H), 7.73 (m, 1H), 7.65 (d, *J* = 8.0 Hz, 1H), 7.53 (d, *J* = 8.0 Hz, 1H), 7.39-7.31 (m, 3H), 5.80 (m, 1H), 5.39 (m, 1H), 5.04 (m, 1H), 2.61 (m,1H), 2.54 (m, 1H).

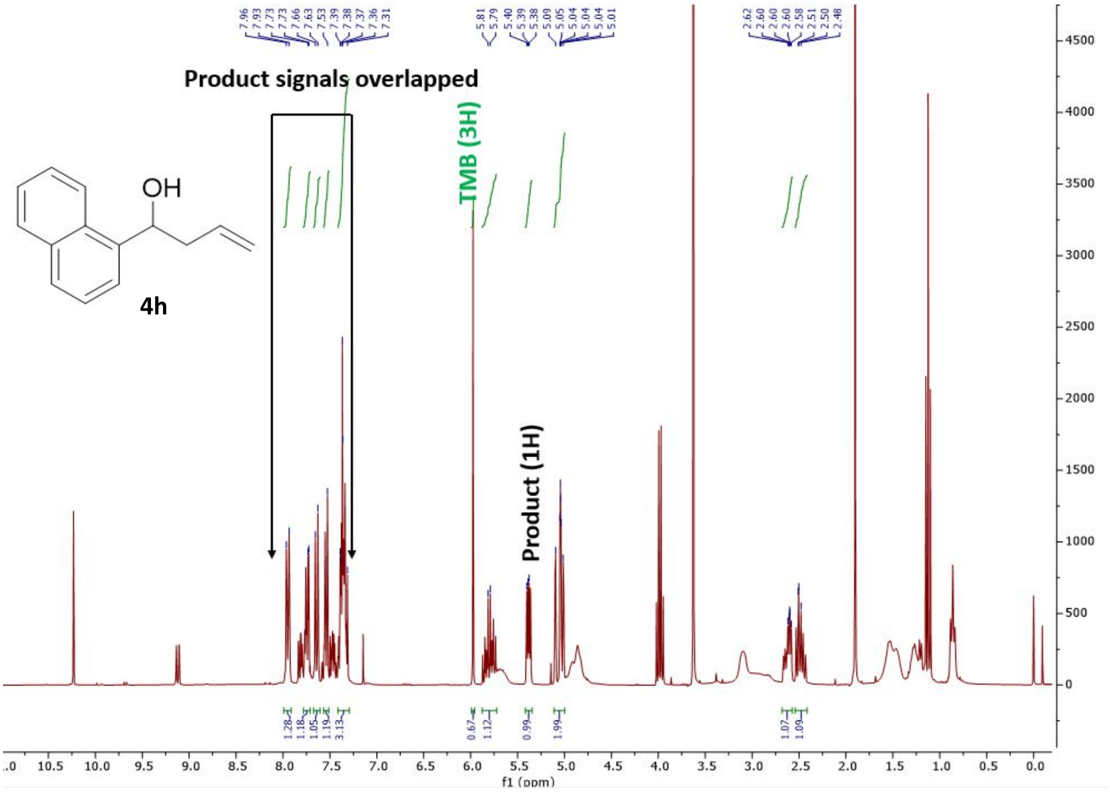

*Figure S47: ^{1}H-NMR spectrum of the crude reaction mixture containing product **4h.***

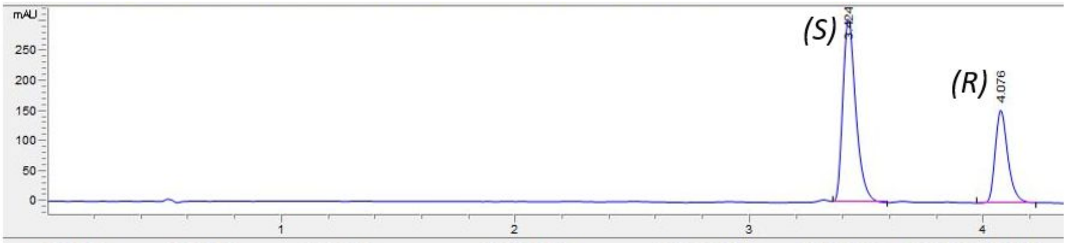

Time	Type	Area	Height	Width	Area%	Symmetry
3.424	BB	1184.6	308.6	0.0588	66.983	0.692
4.076	BB	583.9	155.8	0.0577	33.017	0.739

*Figure S48: SFC-Chromatogram of the crude reaction mixture containing product **4h.** SFC Conditions: IB-Column, gradient 5-30 % MeOH in supercritical CO_2, 10 min.*

(*E*)-1-phenylhexa-1,5-dien-3-ol (4i)[5]:

^{1}H-NMR**:** δ 7.53-7.29 (m, 5H), 6.60 (d, 1H, *J* = 15.0 Hz), 6.25 (dd, 1H, *J* = 15.0 Hz, 6.2 Hz), 5.88-5.85 (m, 1H), 5.21-5.14 (m, 2H), 4.36 (m, 1H), 2.43 (m, 2H).

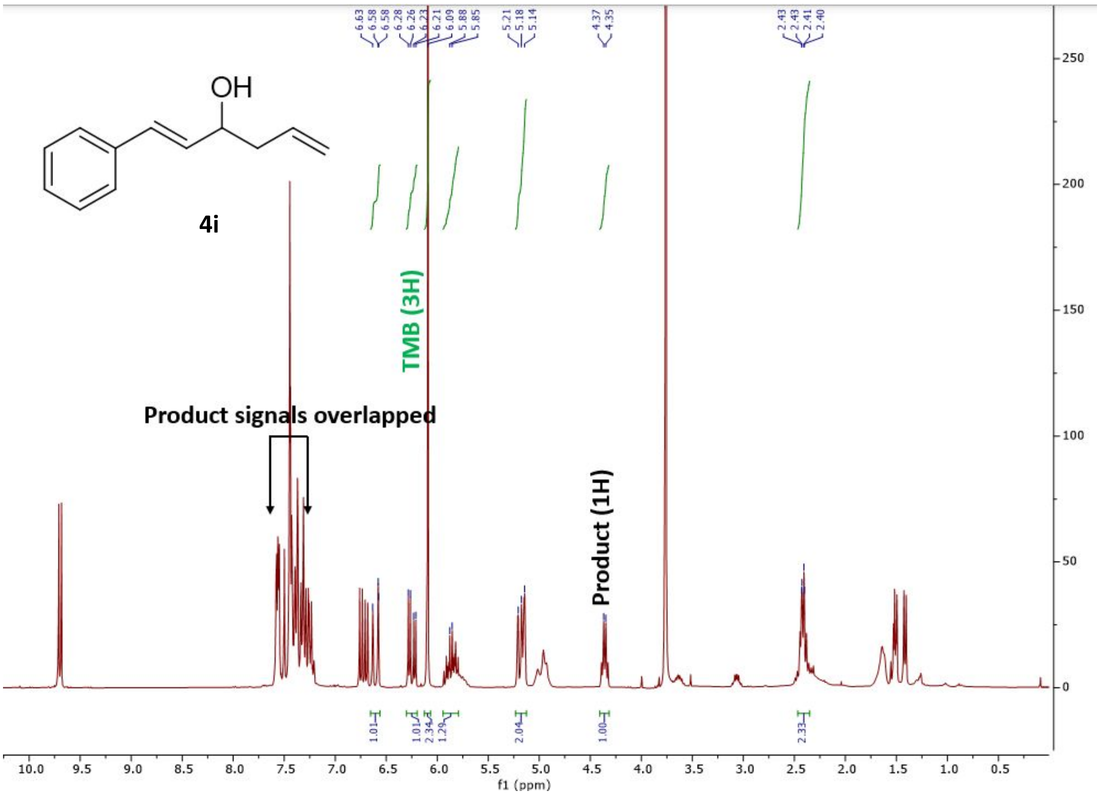

*Figure S49: ^{1}H-NMR spectrum of the crude reaction mixture containing product **4i.***

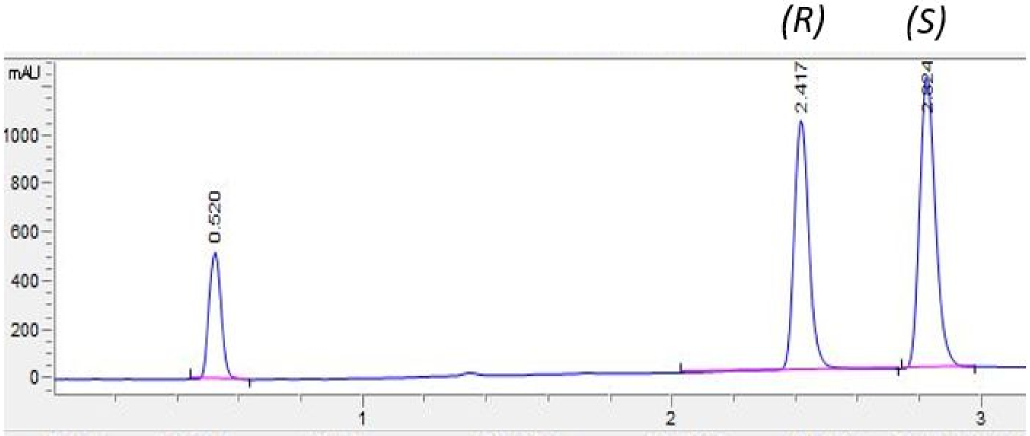

Time	Type	Area	Height	Width	Area%	Symmetry
2.417	BB	3522.1	1032.6	0.0538	38.222	0.847
2.824	BB	4260.3	1200.7	0.0554	46.233	0.795

*Figure S50: SFC-Chromatogram of the crude reaction mixture containing product **4i.** SFC Conditions: IB-Column, gradient 5-30 % MeOH in supercritical CO_2, 10 min.*

18. References

1. Minowa, N.; Mukaiyama, T. Asymmetric Allylation with a New Chiral Allylating Agent Prepared from Tin(II) Triflate, Chiral Diamine, and Allylaluminum. *Bull. Chem. Soc. Jpn.* **1987**, *60* (10), 3697-3704.

2. Kotani, S.; Hashimoto, S.; Nakajima, M., Chiral phosphine oxide BINAPO as a Lewis base catalyst for asymmetric allylation and aldol reaction of trichlorosilyl compounds. *Tetrahedron* **2007,** *63* (15), 3122-3132.

3. González-Gallardo, N.; Saavedra, B.; Guillena, G.; Ramón, D. J. Indium-mediated allylation of carbonyl compounds in deep eutectic solvents. *Appl. Organometallic Chem.* **2021**, *35* (12).

4. Li, N.; Wang, J.; Zhang, X.; Qiu, R.; Wang, X.; Chen, J.; Yin, S.-F.; Xu, X. Strong Lewis acid air-stable cationic titanocene perfluoroalkyl(aryl)sulfonate complexes as highly efficient and recyclable catalysts for C–C bond forming reactions. *Dalton Trans.* **2014**, *43* (30), 11696-11708.

5. Malkov, A. V.; Ramírez-López, P.; Biedermannová, L.; Rulíšek, L.; Dufková, L.; Kotora, M.; Zhu, F.; Kočovský, P. On the Mechanism of Asymmetric Allylation of Aldehydes with Allyltrichlorosilanes Catalyzed by QUINOX, a Chiral Isoquinoline N-Oxide. *J. Am. Chem. Soc.* **2008**, *130* (15), 5341-5348.

6. López-Martínez, J. L.; Torres-García, I.; Rodríguez-García, I.; Muñoz-Dorado, M.; Álvarez-Corral, M. Stereoselective Barbier-Type Allylations and Propargylations Mediated by $CpTiCl_3$. J. *Org. Chem.* **2019**, *84* (2), 806-816.

7. Wang, J. X.; Jia, X. F.; Meng, T. J.; Xin, L. Rapid and solvent-free synthesis of homoallyl or homopropargyl alcohols mediated by zinc powder. *Synthesis-Stuttgart* **2005**, (17), 2838-2844.

8. Yanagisawa, A.; Nakashima, H.; Ishiba, A.; Yamamoto, H. Catalytic Asymmetric Allylation of Aldehydes Using a Chiral Silver(I) Complex. *J. Am. Chem. Soc.* **1996**, *118* (19), 4723-4724.
